# Fluorosulfate-containing pyrazole heterocycles as selective BuChE inhibitors: structure-activity relationship and biological evaluation for the treatment of Alzheimer’s disease

**DOI:** 10.1080/14756366.2022.2103553

**Published:** 2022-07-28

**Authors:** Huan-Huan Li, Chengyao Wu, Shi-Long Zhang, Jian-Guo Yang, Hua-Li Qin, Wenjian Tang

**Affiliations:** a School of Pharmacy, Anhui Medical University, Hefei, China; b School of Chemistry, Chemical Engineering and Life Science, Wuhan University of Technology, Wuhan, China

**Keywords:** Pyrazole, sulphonyl fluoride, cholinesterase inhibitor, SuFEx, anti-amyloid

## Abstract

Novel scaffolds are expected to treat Alzheimer’s disease, pyrazole-5-fluorosulfates were found as selective BuChE inhibitors. Compounds **K1–K26** were assayed for ChE inhibitory activity, amongst them, compound **K3** showed potent BuChE and *h*BuChE inhibition (IC_50_ = 0.79 μM and 6.59 μM). SAR analysis showed that 1-, 3-, 4-subtituent and 5-fluorosulfate of pyrazole ring affected BuChE inhibitory activity. Molecular docking showed that the fluorosulfate increased the binding affinity of *h*BuChE through π-sulphur interaction. Compound **K3** was a reversible, mixed and non-competitive BuChE inhibitor (*K*_i_ = 0.77 μM) and showed remarkable neuroprotection, safe toxicological profile and BBB penetration. *In vivo* behavioural study showed that **K3** treatment improved the A*β*_1 − 42_-induced cognitive impairment, and significantly prevented the effects of A*β*_1 − 42_ toxicity. Therefore, selective BuChE inhibitor **K3** has potential to be further developed as AD therapeutics.

## Introduction

Alzheimer’s disease (AD) is a progressive neurodegenerative disorder, which causes severe impairment of cognitive functionality, neurodegeneration, and even Parkinsonian symptoms, leading the patient to a complete dependence even for accomplishing daily tasks^1^^,^^2^. AD affects 50–60% of people with dementia, which the number of patients will increase from 55 million to an astonishing 151 million by 2050^3^^,^^4^. Clinical evidences demonstrate that AD is a complex disease characterised by progressive memory loss, severe behavioural abnormalities, cognitive impairment and ultimately death. Although the aetiology of AD is not completely known, several factors include acetylcholine (ACh) deficiency, β-amyloid (Aβ) deposits, oxidative stress, dyshomeostasis of biometals and neuroinflammation, have been appointed to play crucial roles in AD onset and progression^5^^,^^6^. However, the current lack of cure is magnifying the problems of AD. AD drugs can only alleviate symptoms of dementia but are not able to halt the progression of the degenerative process.

The cholinergic hypothesis proposes that the degeneration of cholinergic neurons and the associated loss of cholinergic neurotransmission in the cerebral cortex are responsible for the deterioration of cognitive function observed in the brain of AD patients^7^^,^^8^. Acetylcholinesterase (AChE) and butyrylcholinesterase (BuChE) are the two types of cholinesterases (ChEs) for hydrolysis of ACh in brain. AChE is mainly derived from regions of the neural synaptic junction and adult cerebral cortex that express intense activity, while BuChE is mainly derived from glial cells of the brain, maintaining a close spatial relationship of BuChE in glial cells and facilitating BuChE-mediated hydrolysis, thereby regulating local ACh levels, which in turn maintain normal cholinergic function^9–13^. However, the compensatory character of BuChE is greatly noticeable under pathological conditions. In the AChE-knockout mouse model, BuChE was proven to compensate for hydrolysing ACh due to the lack of AChE, thereby maintaining normal cholinergic function^14^. The growing evidence has indicated that, with disease progression, the activity of AChE decreases to 10–15% of normal values in certain brain regions, while BuChE as a compensating enzyme is maintained at the normal or even higher level^15^.

So far, only four ChE inhibitors (rivastigmine, galantamine, donepezil, tacrine) have been approved by FDA for the treatment of AD in clinic. However, they can only provide temporary and incomplete symptomatic relief. In addition, studies have shown that AChE can cause amyloid plaques, and the expression of BuChE is related to Aβ plaques, NFT and cerebral amyloid angiopathy^15–20^. AChE and BuChE are still the most valuable and predominating targets for the discovery of new anti-AD agents. However, selective BuChE inhibitors are mainly pseudoirreversible carbamates and tacrine- or donepezil-based hybrids, but it is limited to discovering new drugs by simply structural modification. Few BuChE-targeted scaffolds were discovered, therefore, search for novel scaffolds with BuChE inhibition was requisite to the treatment of AD^21^^,^^22^.

**Figure 1.**
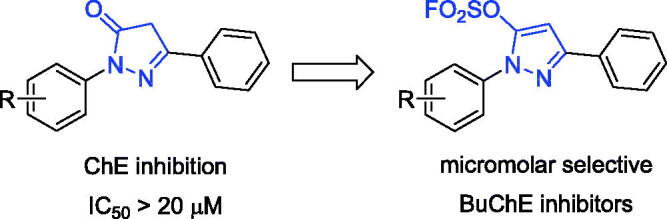
The general design strategy in this study.

Sulphur fluoride exchange (SuFEx) click chemistry enabled covalent linking of modular units through S^VI^ hubs^23^. SuFEx building blocks provided “connective” chemistry that will find structurally versatile scaffolds in chemical biology and drug development, including sultone scaffolds as BuChE inhibitors, sulfamides as novel pesticides^24–30^. Sulphonyl fluorides and fluorosulfates also exhibited excellent biological and pharmacological activities, such as BuChE inhibitors, potent antibacterial agents, cysteine protease SpeB inhibitors, human neutrophil elastase inhibitors, et al.^31–34^. Recently, a series of pyrazolyl fluorosulfates were synthesised through SO_2_F_2_ mediated transformation of pyrazolones^35–39^. The primary screening showed that the 5-fluorosulfate of pyrazole had selective BuChE inhibitory activity compared to the pyrazole, and molecular docking showed that the fluorosulfate of precursor pyrazolone could increase the binding affinity of *h*BuChE through π-sulphur interaction between the sulphur and Trp82 (Figures 1 and S1). In this work, a series of pyrazole-5-sulfofluoridates were screened for their ChE inhibitory activity, analysed for their structure-activity relationship (SAR), and evaluated the preliminary mechanism for treating AD.

## Materials and methods

### Chemistry

Recently, we constructed a class of novel *N*-heterocyclic molecules containing both pyrazole and fluorosulfate as versatile building blocks in the Suzuki coupling reaction and SuFEx click chemistry. Synthetic procedures and characterisation of compounds **K1–K26** were reported in the literature^35^. The purity (relative content) of active compounds was determined by HPLC on an Agilent 1200 instrument (column: Elite, RP-C18, 5 μm, 4.6 × 150 mm) through area normalisation method. TLC was carried out on pre-coated silica gel *F_254_* glass plates with petroleum ether/ethyl acetate (10: 1 → 4: 1). The data in supporting information included characterisation and the copies of representative HPLC spectra of compounds **K1–K26**.

### EeAChE and eqBuChE inhibition assays

According to Ellman's method, assays were carried out on AChE from electric eel (C3389-500UN; Sigma) and BuChE from horse serum (C4290-1KU; Sigma). On a final volume of 500 μL, the experiment was carried out in 48-well plates. EeAChE or eqBuChE, 0.036 U/mL, and 0.1 M pH 8 phosphate buffer were added to each well. At 37 °C, they were pre-incubated for 20 min at various chemical concentrations. Then 0.35 mM acetylthiocholine iodide (ACh; A5751-1G; Sigma) or 0.5 mM butyrylthiocholine iodide (BuCh; 20820–1 G; Sigma) and 0.35 mM 5,5′-ditiobis-2-nitrobenzoico (DTNB; D8130-1G; Sigma) were added. Along with the enzymatic breakdown of ACh or BuCh, the DTNB creates the yellow anion 5-thio-2-nitrobenzoic acid. In a Biotek Synergy HTX Multi-Mode reader, changes in absorbance were detected at 410 nm. Inhibition curves were used to calculate the IC50 values (log inhibitor concentration vs percent of inhibition). The same conditions were used in a control experiment without the inhibitor, and the blank comprised buffer, DMSO, DTNB, and substrate. The final concentration of reactants as follow, compound: 100, 50, 25, 10 and 5 μM; Ach: 0.35 mM; BuCh: 0.5 mM; DTNB: 0.35 mM, which were diluted in 0.1 M pH 8 phosphate buffer.

$$\text{Inhibition}\;\left(\%\right)=\left[\text{control}\;\left({\text{OD}}_{410}\right)-\text{compound}\;\left({\text{OD}}_{410}\right)\right]/\left[\text{control}\;\left({\text{OD}}_{410}\right)-\text{blank}\;\left({\text{OD}}_{410}\right)\right].$$

The IC_50_ values were calculated by SPSS 17.0.

### hAChE and hBuChE inhibition assays

Human AChE (C1682; Sigma), human BuChE (B4186; Sigma), 5,5′-disulfobis (2-nitrobenzoico acid) (DTNB; D8130-1G; Sigma), acetylthiocholine iodide (ACh; A5751-1G; Sigma), and iodobutyrylthiocholine iodide (BuCh; 20820–1 G; Sigma). The enzyme stock solution was produced in gelatine (1 percent deionised water) and then diluted with water to 0.125 unit/mL. ACh and BuCh iodide solutions with a final concentration of 3.75 mM were produced in deionised water. With 0.1 M pH 8 PBS, a DTNB solution (5 μM) was produced. Dissolve the test compound in DMSO to prepare a concentration of 2 × 10^−1 ^M stock solution. The solution was diluted in ethanol to five concentrations of 200, 100, 10, 1 and 0.1 μM, respectively. Measurement was carried out with 96-well plates, add 40 μL buffer, 10 μL test compound at a series of concentrations, and 10 μL AChE/BuChE in sequence. The mixture was then incubated at 37 °C for 10 min. Then add 20 μL DTNB, incubation at 37 °C for 10 min. Finally, add 20 μL of ACh or BuCh and measure the absorbance at 412 nm. For the blank value, 10 μL of ethanol was used instead of the inhibitor solution. The percentage of enzyme activity and the logarithm of compound concentration were used to express the inhibitory activity of compounds. IC50 was calculated by SPSS17.0.

### Kinetic studies of eqBuChE inhibition

The same test settings were used for kinetic studies, with six concentrations of substrate (0.1‒1 mM) and four concentrations of inhibitor (0‒1.6 µM). The effect of different doses of compound **K3** on BuChE catalytic activity at 37 °C was also investigated. The final concentration of enzyme was changed (0‒0.18 U/mL) under the same test conditions as indicated in the assay methodology. For the assessment of reversible and irreversible inhibitor binding at enzyme, different concentrations of compound **K3** (0, 0.4, 0.8, and 1.6 µM) were utilised.

### Molecular docking study

The binding mechanisms of active compound to BuChE enzyme active sites were discovered using a structure-based in silico method. The Discovery Studio Client v18.1.0 (DS) CDOCKER was used to explain the SAR of series compounds and to guide the development of more effective and tangible BuChE inhibitors. As a template, the ligand binding to the crystal structure of *h*BuChE (PDB ID: 6QAA) was used. To assure the target enzyme's integrity, it was produced using DS's Prepare Protein. Full Minimisation of the Small Molecular in DS was used to prepare the ligand. Then, using CDOCKER, the title compounds were docked into the active site of the protein. The view results of molecular docking were extracted after the program running end, each docking result was analysed for interaction and their different pose. The most stable poses were those with the lowest -CDOCKER_INTERACTION_ENERGY values and then picked them to analysize the binding interactions with target enzyme visualised.

### Cytotoxicity assays

Human hepatoblastoma cells HepG2 and human normal liver cells LO2 cells were cultured at 37 °C in DMEM containing 10% foetal bovine serum, 100 U/mL penicillin, and 100 mg/mL streptomycin in a humidified incubator with 5% CO_2_. The methyl thiazolyl tetrazolium (MTT) assay was used to assess cell cytotoxicity. In a 96-well plate, HepG2 cells and LO2 cells were injected at 1 × 10^4^ cells per well. The cells were treated with various substances that were diluted in DMEM for 24 h after being cultivated for 24 h. The cells were then treated for 4 h with 20 µL 0.5 mg/mL MTT reagent. The cell culture was withdrawn after 4 h, and 150 µL DMSO were added to dissolve the formazan. At 492 nm, the optical density was observed (OD492). Three independent tests were used to calculate cell viability.

Cell viability (%)=compound (OD492)/blank (OD492)×100%.

Blank: cultured with fresh medium only.

Compound: treated with compounds or donepezil.

### Neuroprotection assay

PC12 cells were distributed at a density of 1 × 10^4^ cells per well into 96-well microtiter plates. At time zero, cells were treated with a range of compounds **K3** concentrations (1‒25 µM) and kept for 3 h after being incubated overnight. The media were then changed with new media containing the medication as well as a cytotoxic stimulus in the form of 100 µM H_2_O_2_, which was left for another 24 h. The MTT assay was used to determine cell viability after 24 h. In the cells, 20 µL of 0.5 mg/mL MTT reagent was applied and incubated for 4 h. The cell culture was removed after 4 h, and 150 µL DMSO was added to dissolve the formazan. The optical density was measured at 492 nm (OD492) on the Biotek Synergy HTX Multi-Mode reader. Results were adjusted considering OD measured in the blank.

### PAMPA-BBB penetration assay

Based on prior work^40^^,^^41^, a parallel artificial membrane permeation assay (PAMPA) for the blood-brain barrier (BBB) was used to determine the ability of the test chemical to penetrate into the brain. Six commercial medicines were purchased from Aladdin Reagents to validate the methodology. Energy Chemical provided the DMSO and dodecane. Avanti Polar Lipids provided the porcine brain lipid (PBL). Millipore provided the donor 96-well filter microplate with a PVDF membrane (pore size 0.45 mm) and the acceptor indented 96-well microplate. Corning Inc. provided the 96-well UV plate (COSTAR). DMSO was used to dissolve commercial medications and test compounds at a concentration of 20 mg/mL. They were then diluted 200 times in a solution of PBS (pH 7.4 ± 0.1)/EtOH (70/30, v/v) to achieve a final concentration of 100 µg/mL. The donor microplate's fifilter membrane was coated with 4 µL of PBL in dodecane (20 mg/mL). The donor wells were then filled with 200 µL of diluted chemical solution and 300 µL of PBS/EtOH (70/30, v/v). To make the membrane contact with buffer solution, the donor fifilter plate was carefully positioned on top of the acceptor plate to form a “sandwich” assembly. At 25 °C, the sandwich was left undisturbed. The donor plate was carefully removed after 18 h of incubation, and the concentrations of test chemicals in the donor and acceptor wells were quantified using a UV plate spectroscopic reader (PerkinElmer VICTOR Nivo, Finland). Each sample was analysed at three wavelengths, in at least three independent experiments, in four wells.

*P*_e_, permeability, refers to the ability of compounds to permeate the membranes through passive diffusion in the blood-brain barrier penetration assays.

$$P_{e}=\frac{-\text{ ln }(1-C_{\mathrm{acceptor}}/C_{\mathrm{equilibrium}})}{\mathrm{aera}\;\times \;(1/V_{D}+\;1/V_{A})\;\times \;\mathrm{time}}$$

$$C_{\mathrm{equilibtium}}=\frac{C_{D}\;\times \;V_{D}\;+C_{A}\;\times \;V_{A}}{V_{D}\;+{\;V}_{A}}$$

### In vivo acute toxicity evaluation

A total of 20 mice weighing 20‒25 g (F: M = 1: 1) were randomly allocated into two groups: control (*n* = 10) and experimental (*n* = 10). In a mixture of DMSO, PEG 400, and physiological saline (10/50/40, v/v/v), compound **K3** was suspended. On the first day, the mice were intragastrically given the vehicle or test substance **K3** 0.45 g/kg after fasting for 8‒12 h. For two weeks, the mice's behaviour, appearance, and body weight changes were examined and documented. GraphPadPrism8.0 software was used to compare and summarise the body weights of the mice in the control and experimental groups.

### In vivo hepatotoxicity evaluation

Compound **K3** was suspended in a mixed solution of DMSO, PEG 400, and normal saline (10/50/40, v/v/v) to assess in vivo hepatotoxicity in male ICR mice (20‒25 g), which were also divided into blank and experimental groups. Mice were fasted for 24 h. Intragastrically, the combination was given at a dose of 30 mg/kg body weight with the same quantity of vehicle (po). With comparable kits, the activities of alanine aminotransferase (ALT) and aspartate aminotransferase (AST), both markers of liver damage, were assessed (EF551 and EF550 for ALT, EH027 and EH548 for AST). A biochemical analyser was used to process the data (Hitachi 7020, Japan). Mice were slaughtered and livers were obtained for immunohistochemical morphological evaluation after the last sample of postglobular blood. We isolated two 3-mm sections of each liver from the hilum to the edge of the left lateral lobe using an ultra-thin semi-automatic microtome (LeicaRM2245, Germany), immediately placed them in 10% buffered formaldehyde, fixed them for 2 days, and embedded them in paraffin blocks using a paraffin-embedding station (LeicaEG1150H, Germany). Subsequently, five µM sections were prepared from these paraffin sections, deparaffinized, stained with haematoxylin and eosin (HE) or using the periodic acid-Schiff glycogen staining method.

### Animal studies

The National Institutes of Health Guide for the Care and Use of Laboratory Animals was followed for all experiments. The Animal Care and Use Committee of Anhui Medical University adopted the Measures for the Care and Treatment of Laboratory Animals. In Anhui Medical University's animal centre, male ICR mice were employed (Hefei). Male mice (18‒24 g) aged 6‒8 weeks, 10 mice per cage, room temperature 22 ± 2 °C, light/dark (12:12 h) cycle Prior to testing, these animals had access to food and water. Throughout all of the experiments, the room's ambient temperature and relative humidity (50%) remained constant. Behavioural tests: MWM was used to test cognitive function, and mice were chosen at random for behavioural tests. 8‒10 animals were used in each study group. The experimental time was from 08: 00 to 14: 00, and the mice were sacrificed by cervical dislocation shortly after the experiment ended. Positive and test chemicals were suspended in a combination of DMSO and 0.5% sodium carboxymethylcellulose (1/99, V/V) before the experiment for the Aβ_1–42_ oligomerization damage test, and 40 male mice were randomly allocated into five groups: (i) blank control group (po); (ii) saline (icv) + vehicle (proper amount, po); sham-operation group; (iii) oligomerized Aβ_1–42_ peptide (10 µg/mouse, icv) + vehicle (proper amount, po); model group; (iv) oligomerized Aβ_1–42_ peptide (10 µg/mouse, icv) + donepezil (15 mg/kg, po), donepezil group, (v) oligomerized Aβ_1–42_ peptide (10 µg/mouse, icv) + **K3** (10 mg/kg, po), **K3** group. A*β*_1–42_ aggregation was induced by dissolving the A*β*_1–42_ peptide in DMSO as a stock solution of 5 mM and incubating it in saline at a final concentration of 2 mg/mL for 24 h at 37 °C.

The mice participated in a water maze experimental behavioural investigation on days 10–15, which included a 5-day learning and memory training and a test evaluation on day 6. MWM is made up of a water-filled pool (gray, circular, 1.20 m in diameter, 0.60 m in height) and a platform that can be adjusted in height and moved. Using a video tracking system, the pool was divided into four equal quadrants (compass position: NE, NW, SE, SW) (SMART, version 3.0; Panlab, Spain). To prevent animals from jumping out, the pool was filled with water up to 48 cm below the edge, and the water temperature was kept at 22 ± 1 °C. The escape platform was made of transparent plexiglass (11 cm in diameter and 47 cm in height) and was soaked 1 cm below the water level in a fixed position (the middle of the northwest quadrant, that is, the target quadrant). The pool is in a larger area with no light shadow on the pool water surface and four reference objects on the pool wall with distinct geometric designs. During the training, the mice were randomly placed in the water facing the pool wall from one of four quadrants (NE, NW, SE, SW), with the platform in the southeast quadrant, and each experimental mouse swam for a total of 60 s to find the hidden platform. If the mouse was unable to locate the platform in the pool or climb it within 60 s of entering the water, the mouse was instructed to stand on the platform for 15 s. The average speed, the time to reach the hidden platform (i.e., escape latency), the distance to reach the hidden platform, and the distance in the target (NW) area were all recorded. The platform was removed from the pool on day 6 (24 h following the last training session) and a probing trial (Drogoff test) was conducted. Each mouse was allowed to swim once, and if the previous platform position was not found within 60 s, a latency score of 60 s was given to measure the latency to first cross the previous platform position (i.e., the target area), the number of times it crossed the target area, the time spent in the target NW quadrant, the total distance, the distance spent in the NW quadrant, the entry into the NW quadrant, and the mean speed, and compared across experimental groups.

In the Aβ_1–42_ oligomeric damage experiment, all mice were sacrificed after the behavioural research was completed, and the brains were collected to assess the total content of Aβ_1–42_ using a mouse ELISA kit. Each brain tissue sample was thoroughly homogenised in a grinder with 10 times PBS (pH = 7.4 ± 0.1) before being centrifuged for 5 min at 5000×*g*. The supernatant was separated for use. The detection technique followed the directions exactly, and the standard curve was created. Brain tissue A*β*_1–42_ content was calculated according to the linear regression equation. All values were expressed as mean ± SEM using GraphPadPrism8.0 software.

### Statistical analysis

Data are reported as mean ± SEM of at least three independent experiments and data analysis was performed with GraphPad Prism 8 software.

## Results and discussion

### Chemistry

**Scheme 1.**
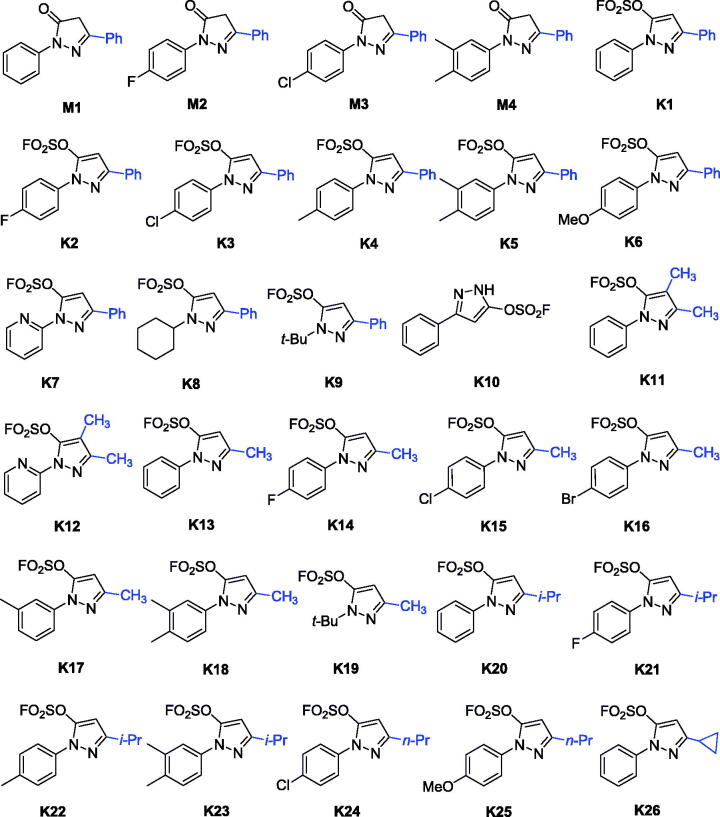
Chemical structures of compounds **M1–M4** and **K1–K26**.

Recently, a class of novel *N*-heterocyclic molecules containing both pyrazole and fluorosulfate was constructed as versatile building blocks in the Suzuki coupling reaction and SuFEx click chemistry^35^. The novel fluorosulfate-containing pyrazole heterocycles (compounds **K1–K26** as shown in Scheme 1) were synthesised through the reaction of pyrazolones with SO_2_F_2_ under 1.5 equivalents of DIPEA in CH_2_Cl_2_ in good to excellent yields.

### Inhibitory activity against AChE and BuChE

The materials **M1–M4** and pyrazole-5-fluorosulfates **K1–K26** were evaluated for their activity with *Electrophorus electricus* AChE (EeAChE) and equine BuChE (eqBuChE), using modifified Ellman’s method. The IC_50_ values were obtained and compared to the reference rivastigmine, and donepezil, which is a selective AChE inhibitor that simultaneously binds to catalytic active and peripheral anionic sites (PASs)^42^. The IC_50_ values of all tested compounds against *ee*AChE and eqBuChE are summarised in Table 1.

**Table 1.** Inhibitory activities against *ee*AChE and eqBuChE of compounds **M1–M4** and **K1–K25** (IC_50_, µM or % inhibition at 20 µM).^a^

	(IC_50_, µM or % inhibition at 20 µM)	
Compound	AChE^b^	BuChE^c^
**M1**	8.8 ± 2.6%	*na*
**M2**	37.0 ± 3.3%	27.7 ± 11.7%
**M3**	26.9 ± 1.0%	25.4 ± 5.4%
**M4**	10.7 ± 1.9%	44.1 ± 13.8%
**K1**	33.6 ± 1.6%	2.28 ± 0.64
**K2**	27.5 ± 2.7%	1.73 ± 0.62
**K3**	26.3 ± 1.4%	0.79 ± 0.32
**K4**	25.9 ± 0.9%	2.35 ± 1.02
**K5**	24.4 ± 2.1%	2.23 ± 0.31
**K6**	25.7 ± 1.5%	47.9 ± 1.9%
**K7**	19.39 ± 0.01	9.89 ± 0.87
**K8**	25.1 ± 1.2%	5.84 ± 0.87
**K9**	10.1 ± 0.4%	30.9 ± 1.5%
**K10**	32.0 ± 1.2%	6.92 ± 1.41
**K11**	7.2 ± 1.0%	45.2 ± 1.8%
**K12**	42.8 ± 0.8%	20.7 ± 1.7%
**K13**	37.5 ± 1.4%	9.16 ± 0.53
**K14**	48.2 ± 1.0%	5.50 ± 0.09
**K15**	45.2 ± 1.3%	5.26 ± 0.39
**K16**	37.2 ± 0.7%	7.64 ± 0.41
**K17**	33.4 ± 1.3%	19.18 ± 0.69
**K18**	8.1 ± 0.5%	3.31 ± 0.70
**K19**	*na*	*na*
**K20**	41.6 ± 0.7%	11.25 ± 0.11
**K21**	7.79 ± 0.07	6.90 ± 0.42
**K22**	2.60 ± 0.27	17.99 ± 0.34
**K23**	21.4 ± 1.1%	3.98 ± 0.09
**K24**	41.8 ± 0.2%	4.59 ± 0.06
**K25**	10.3 ± 0.6%	3.47 ± 0.04
**K26**	4.82 ± 0.07	14.36 ± 1.08
Donepezil	0.071 ± 0.02	9.66 ± 0.60
Rivastigmine	16.13 ± 1.21	0.063 ± 0.03

^a^Each IC_50_ value is the mean ± SEM from at least three independent experiments; ^b^AChE from electric eel; ^c^BuChE from horse serum; ^d^*na*, no inhibitory activity (%) against either EeAChE or eqBuChE at 20 µM.

Enzymatic assays revealed that all pyrazole-5-fluorosulfates **K1–K25** showed inhibitory activities against AChE and BuChE except for compound **K19**, while their precursors 1,3-disubstituted-*1H*-pyrazol-5(*4H*)-ones **M1–M4** showed weak inhibitory activity against AChE and BuChE (IC_50_ > 20 μM). Compound **K22** showed selective AChE inhibition (IC_50_ values for AChE and BuChE, 2.60 and 17.99 μM, respectively), while the other exhibited moderate to strong inhibitory activity against showing selectivity towards BuChE. It was obvious from the data that compound **K3** exhibited the best inhibitory activity against BuChE with IC_50_ value of 0.79 μM. Inspection of the chemical structures, it can be concluded that the BuChE inhibitory activity was affected by the substituent groups at the 1-, 3- or 4-positions of pyrazole fluorosulfates (Table 1). From Table 1, it is intuitive that the substituent of 1-aryl ring and 3-positon at the pyrazole ring plays important role on the activity.

### SARs of pyrazole-5-fluorosulfates for BuChE inhibition

As shown in Scheme 1 and Table 1, pyrazole-5-fluorosulfate derivatives exhibited selective BuChE inhibitory activity except for compound **K19**. The structure-activity relationship (SAR) was further analysed. Firstly, the substituent at 1-phenyl ring of pyrazole-5-fluorosulfate affects the BuChE inhibitory activity: (i) the order of substituent at 4-position: –Cl > –F > –Br > –H > –CH_3_, such as **K3** (–Cl, 0.79 μM) > **K2** (–F, 1.73 μM) > **K1** (–H, 2.28 μM) > **K4** (–CH_3_, 2.35 μM) for 3-phenyl, **K15** (–Cl, 5.26 μM) > **K14** (–F, 5.50 μM) > **K16** (–Br, 7.64 μM) > **K13** (–H, 9.16 μM) for 3-methyl, **K21** (–F, 6.90 μM) > **K20** (–H, 11.25 μM) > **K22** (–CH_3_, 17.99 μM) for 3-isopropyl; (ii) 3,4-diMe > mono-Me: **K5** (–diMe, 2.23 μM) > **K4** (4-Me, 2.35 μM) for 3-phenyl, **K18** (–diMe, 3.31 μM) > **K17** (3-Me, 19.18 μM) for 3-methyl, **K23** (–diMe, 3.98 μM) > **K22** (4-Me, 17.99 μM) for 3-isopropyl; (iii) when 1-phenyl was substituted by smaller group (*t*-Bu), BuChE inhibition decreased, such as **K1 **>** K9**, **K13 **>** K19**.

Secondly, the effect of substituent at 3-position of pyrazole on BuChE inhibition was observed: (i) –phenyl > –CH_3_ > –*i*-Pr, such as **K1** (2.28 μM) > **K13** (9.16 μM) > **K20** (11.25 μM) for 1-phenyl, **K2** (1.73 μM) > **K14** (5.50 μM) > **K21** (6.90 μM) for 4-F-phen-1-yl, **K3** (0.79 μM) > **K15** (5.26 μM) for 4-Cl-phen-1-yl, **K4** (2.35 μM) > **K22** (17.99 μM) for 1–4-Me-phenyl, **K5** (2.23 μM) > **K18** (3.31 μM) > **K23** (3.98 μM) for 1–3,4-diMe-phenyl, **K9** (30.9%) > **K19** (no activity) for 1-*t*-butyl; (ii) when the substituent at 3-position was *n*-propyl, compounds exhibited potent BuChE inhibitory activity (IC_50_ = 4.59 and 3.47 μM for **K24** and **K25**, respectively); (iii) cyclisation of the *i*-propyl (IC_50_ = 11.25 μM for **K20**) led to decrease the activity (IC_50_ = 14.36 μM for **K26**).

**Figure 2.**
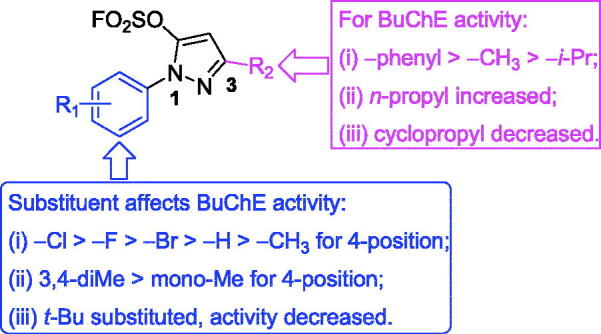
SARs of the pyrazole-5-sulfofluoridate on BuChE inhibitory activity.

Finally, the multi-substituent of pyrazole ring led to decrease BuChE inhibitory activity, for example, inhibitory rates of compounds **K11** and **K12** were 45.2% and 20.7%, respectively. All in all, the SAR of pyrazole-5-sulfofluoridate scaffold is illustrated in Figure 2.

### Inhibition of hBuChE and hAChE

In order to determine the potency and selectivity of compounds **K3** for the human enzymes, ChE inhibitory activity was detected on *h*AChE and *h*BuChE. As shown in Table 2, Compared to the positive control rivastigmine, compound **K3** showed stronger inhibitory effect on *h*BuChE (IC_50_ = 6.59 µM) and have inhibitory effect on *h*AChE (31.65% inhibitory rate at 20 μM). Hence, compound **K3** was found as a selective *h*BuChE inhibitor.

**Table 2.** Inhibitory activity on *h*AChE and *h*BuChE.^a^

Compound	IC_50_, µM (or inhibition% at 20 µM)	
	*h*AChE^b^	*h*BuChE^c^
Donepezil	0.008 ± 0.004	12.42 ± 0.90
Rivastigmine	10.45 ± 2.95%	7.77 ± 2.92
**K3**	31.65 ± 5.74%	6.59 ± 3.54

^a^Each IC_50_ value is the mean ± SEM from at least three independent experiments; ^b^*h*AChE from recombinant human AChE (*h*AChE); ^c^*h*BuChE from human serum.

### Kinetic study of eqBuChE inhibition

**Figure 3.**
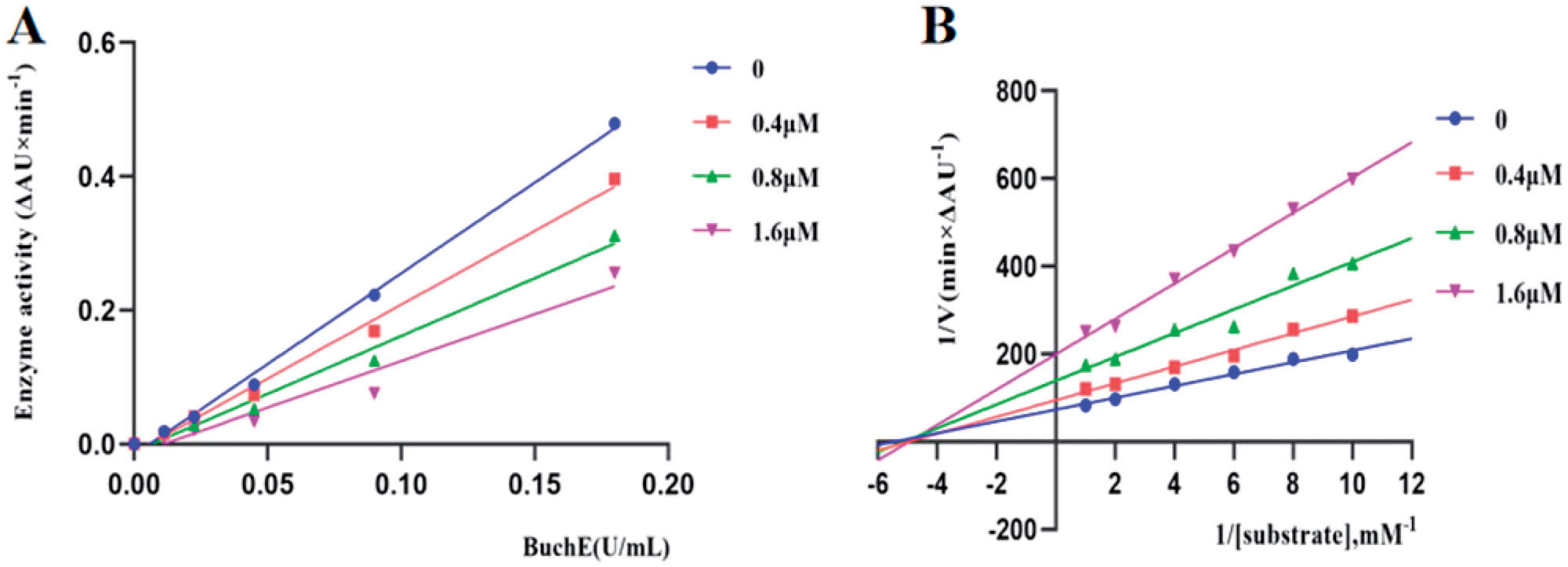
Relationship between eqBuChE inhibition and various concentrations of **K3** (A). Lineweaver‒Burk plots of eqBuChE inhibition kinetics of **K3** (B).

Potent inhibitor **K3** was subjected to enzyme kinetics analysis to determine the kinetics of BuChE inhibition^43–45^. As shown in Figure 3(A), the plots of the remaining enzyme activity versus the concentration of enzyme (0, 0.01125, 0.0225, 0.045, 0.090 and 0.18 U/mL) in the presence of different concentrations of compound **K3** for the catalysis of butyrylcholine gave a series of straight lines. Concentrations of compound **K3** (0, 0.4, 0.8, 1.6 μM) were used respectively, for the determination of reversible as well as irreversible binding of inhibitors at enzyme. In case of compound **K3**, all the lines intersected at the same point. With the inhibitor concentration increased, the slope of the lines decreased, which indicated that compound **K3** was reversible BuChE inhibitors. Kinetic studies of eqBuChE inhibition were performed with the same test conditions, using six concentrations of substrate (0.1‒1 mM) and four concentrations of inhibitor (0‒1.6 μM). The concentration effect of compound **K3** on the activity or the catalysis of BuChE at 37 °C was also studied. Assay conditions were the same as described in assay protocol except that the final concentration of enzyme was varied (0‒0.18 U/mL). As shown in Figure 3(B), overlaid reciprocal Lineweaver-Burk plots confirmed it is a typical non-competitive inhibitors because all lines intersect in the second quadrant. And the dissociation constant *K*_i_ of compound **K3** from the Lineweavere-Burk secondary plots was estimated to be 0.77 μM.

### Molecular docking of compound K3

**Figure 4.**
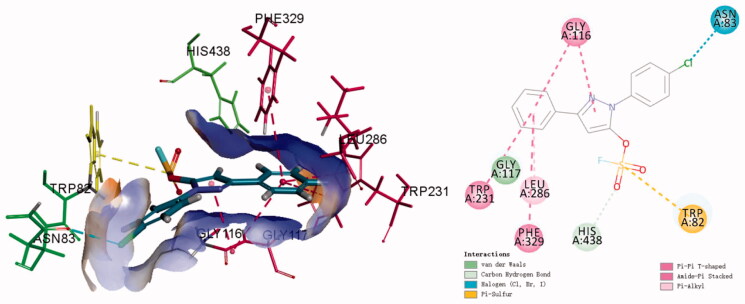
3 D diagram (A) and 2 D diagram (B) of compounds **K3** into *h*BuChE (PDB: 6QAA) performed respectively. Active site residues of *h*BuChE are presented as sticks with carbon atoms represented in light green. The green dashed lines represent hydrogen bonds, the light blue dashed lines represent halogen interaction bonds and the light pink dashed line represents π-alkyl interaction.

The fluorosulfate of pyrazolone was observed to increase the binding affinity with *h*BuChE in Figure S1, however, a re-docking protocol was carried out to understand the binding modes of **K3** targeting *h*BuChE in detail. As shown in Figure 4(A,B), compound **K3** could insert into the binding groove of *h*BuChE, forming multiple interactions *via* π-π interaction between the benzene ring and Gly116 and Trp231 and Phe329, halogen bond between the chlorine and Asn83. Moreover, the fluorosulfate (SO_2_F) moiety could form π-Sulphur interaction with Trp82 and carbon hydrogen bond interaction with His438, which is consistent with the result in Figure S1.

### Cytotoxicity assays

**Figure 5.**
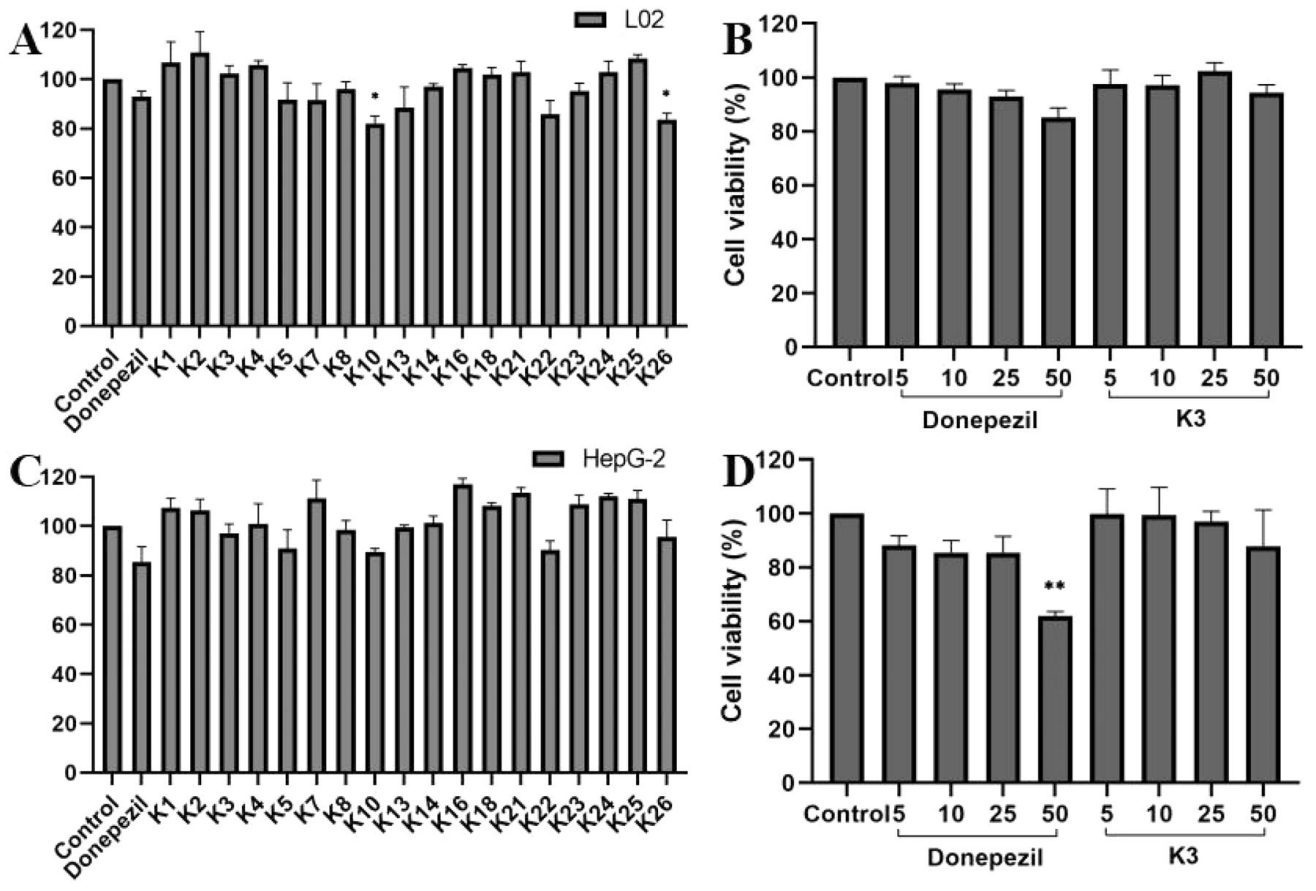
The cytotoxic effect of active compounds on L02 (A) cells and HepG2 (C) cells for 24 h was determined at a concentration of 25 µM, and untreated cells were used as controls. L02 (B) cells and HepG2 (D) cells were treated with donepezil and **K3** at concentrations ranging from 1 to 50 µM for 24 h. Untreated cells were used as controls. Results were expressed as a percentage of cell survival versus untreated cells (control) and as mean ± SEM (*n* = 3, *p < 0.05, **p < 0.01, ***p < 0.001 vs control group).

The cytotoxicity of active compounds was tested using human normal hepatocyte L02 and human hepatoblastoma HepG2 by the 3–(4,5-dimethylthiazol-2-yl)-2,5-diphenyltetrazolium (MTT) assay^24^^,^^46^. As shown in Figure 5(A,C). At the concentration of 25 μM, the cell viability of all compounds was greater than 80% for HepG2 and LO2 cells, indicating that compound **K3** has a wide range of therapeutic safety. On the basis of enzyme inhibition activity and cytotoxicity, compound **K3** was selected as the representative. As shown in Figure 5(B,D), the cell survival rate of compound **K3** was maintained at 94.4% and 87.9% even at 50 μM. The results showed that compound **K3** had broad therapeutic safety against L02 and HepG2 cells.

### Neuroprotective study

**Figure 6.**
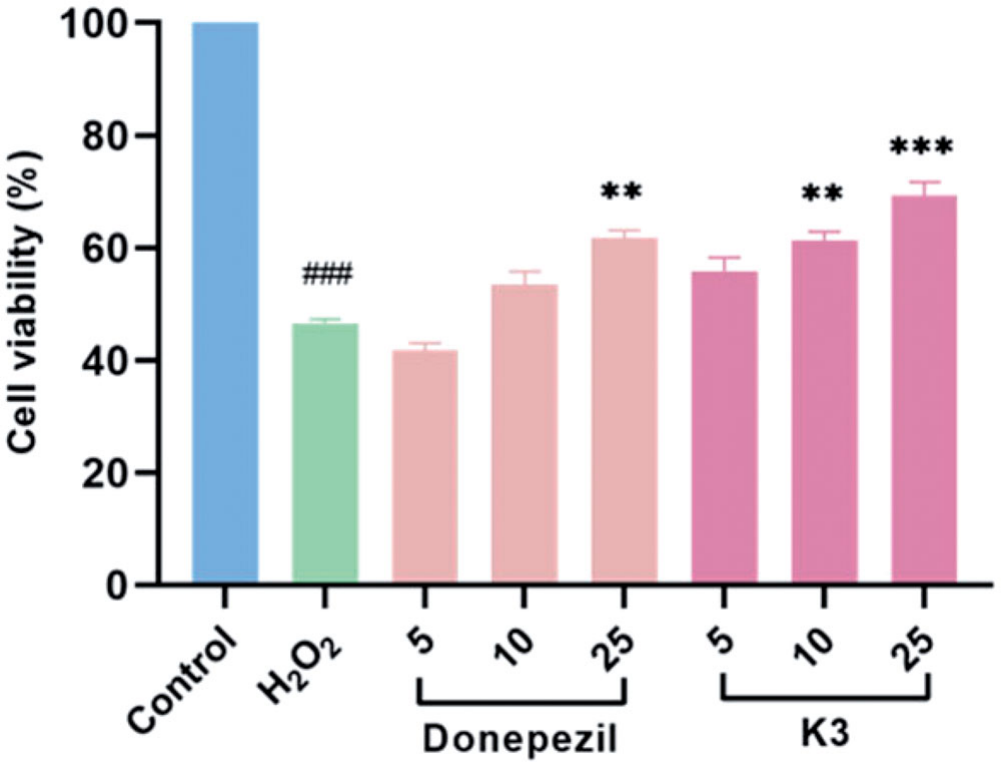
Neuroprotective effect of **K3** (0–25 µM) against H_2_O_2_-induced (100 µM) PC12 neurons injury for 24 h (cells are given **K3** and incubated for 3 h and then given H_2_O_2_ peroxide to model). Results represent mean ± SEM (*n* = 3, ^###^*p* < 0.001 vs control group; **p < 0.01, ***p < 0.001 vs model group).

The protective effects of compound **K3** against free radicals damage were assessed by measuring the ability of the compound to protect against H_2_O_2_ injury^47^^,^^48^. After 100 µM H_2_O_2_ exposure, cell viability as determined by MTT reduction was obviously decreased to 46.5% (*p* < 0.01 vs control), manifesting high sensitivity to H_2_O_2_-induced injury. Compound **K3** showed protective effects in a dose-dependent manner against H_2_O_2_-induced PC12 cell injury (Figure 6). At a concentration of 25 µM, compound **K3** showed neuroprotective effects and was slightly stronger than the positive control donepezil, with cell viability of 69.3%. When the concentration was reduced to 5 µM, the cell viability decreased to 55.9%. It results showed that compound **K3** had a good protective effect on H2O2-induced PC12 cell damage.

### PAMPA-BBB penetration assay

**Figure 7.**
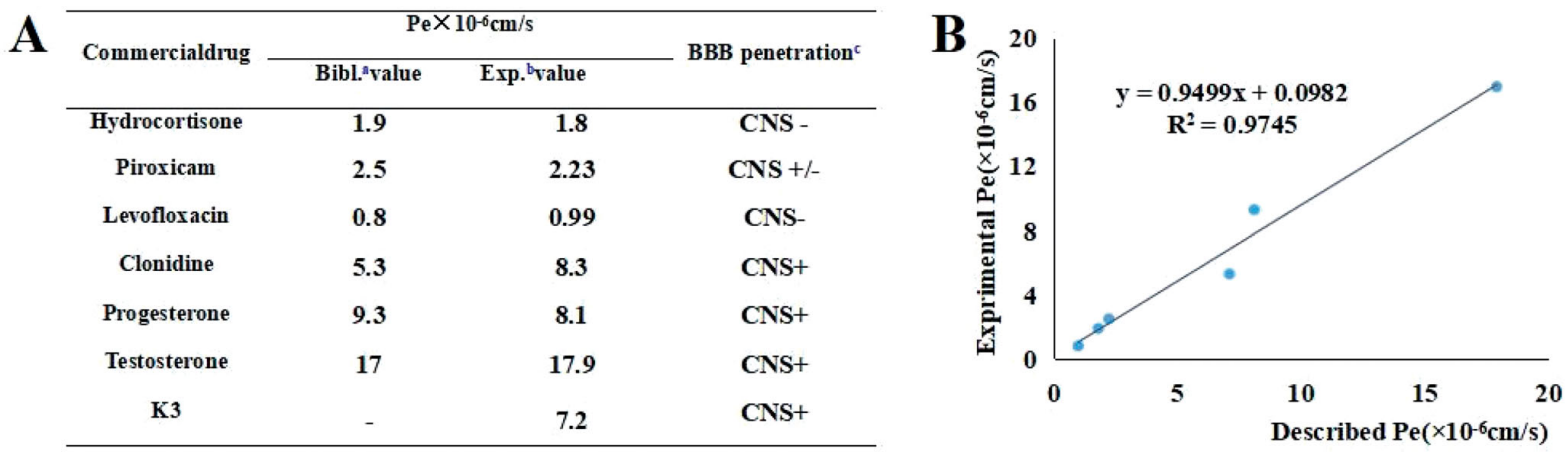
PAMPA-BBB penetration study of **K3**. (A) Results of the PAMPA-BBB assay for six commercial drugs used in the experimental procedure validation and **K3**. (B) Linear correlation presenting experimental versus bibliographic data of commercial drugs. ^a^Bibl. values are reported data from the reference; ^b^All tests were obtained from three independent experiments; ^c^“CNS +” (high BBB permeation): *P*_e_ (× 10^‒6 ^cm/s) > 4.0; “CNS +/‒” (uncertain BBB permeation): *P*_e_ (× 10^‒6 ^cm/s) from 2.0 to 4.0; “CNS ‒” (low BBB permeation): *P*_e_ (× 10^‒6 ^cm/s) < 2.0.

It is essential to evaluate the BBB penetration capacity of active compounds into the central nervous system as the therapeutic target of the anti-AD agent^49^^,^^50^. PAMPA (parallel artificial membrane permeation assay) is an *in vitro* model of passive diffusion, which was used to predict BBB penetration. Hence, BBB penetrating ability of **K3** was estimated using PAMPA-BBB model. To validate the experimental procedure, we chose six commercial drugs with reported values. A plot of experimental versus bibliographic data presented a good linear correlation, *P*_e_ (exp.) = 0.9499 *P*_e_ (bibl.) + 0.0982 (*R*^2^ = 0.9745) (Figure 7(B)). **K3** was tested following this procedure, and the results in Figure 7(A) showed that permeability values of tested compounds are above 4 × 10^−6 ^cm/s, indicating the potential BBB penetration of **K3**.

### In vivo acute toxicity evaluation

**Figure 8.**
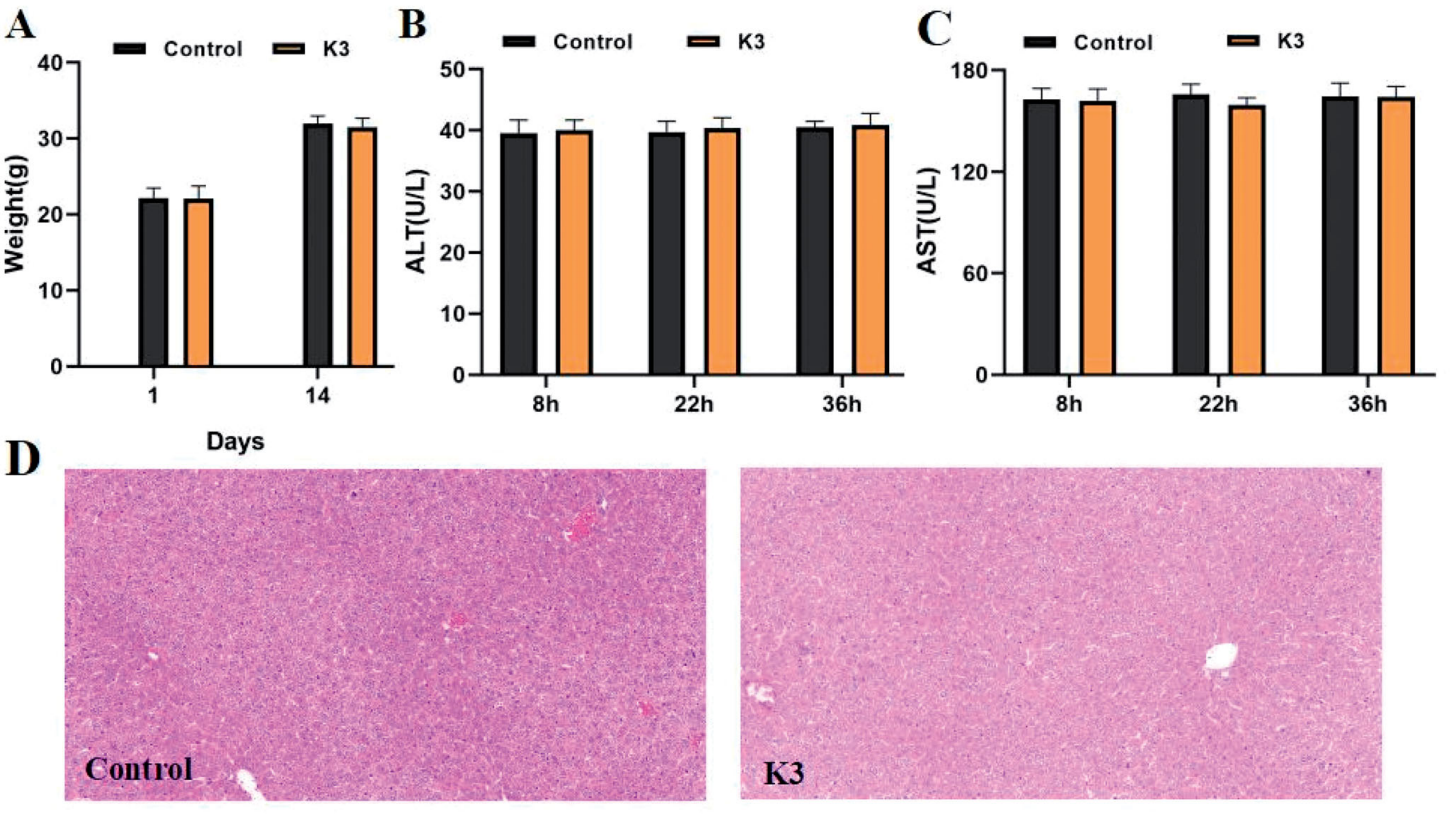
Results of in vivo acute toxicity evaluation. (A) Body weight of ICR mice (g) ‒ measurement time point (day); (B) ALT activity at 8, 22 and 36 h after single dosing (control) or **K3**; (C) AST activity at 8, 22 and 36 h after single dosing (control) or **K3**; (D) Histomorphological observation of mouse liver treated with **K3** vector (control); The HE-stained field was 100 µM.

The in vivo toxicity of compound **K3** was assessed using ICR mice in a single-dose acute toxicity assay. The rats' general condition was satisfactory after intragastric treatment of compound **K3** (1.0 g/kg), with no noticeable changes in appearance or activity. The body weight of mice in the vehicle group and **K3** groups increased throughout the 14 days prior to treatment, as shown in Figure 8(A), although the differences in body weight changes were not significant, showing that compound **K3** was well tolerated at high dosages (1.0 g/kg). As shown in Figure 8(B,C), serum ALT and AST levels were directly proportional to the degree of liver injury. No significant difference was found (*p* > 0.05) between the vehicle group and the **K3** group at each time point, nor within the **K3** group at each time point. The hepatotoxicity of compound **K3** was detected morphologically using immunohistochemistry. Figure 8(D) displays the paraffin sections of the control group and the compound **K3** (30 mg/kg) after 36 h of administration. The findings demonstrated that compound **K3** had great safety in vivo since there was no core necrosis or evident steatosis in and around the intermediate zone around the hilum.

### Behavioural studies

**Figure 9.**
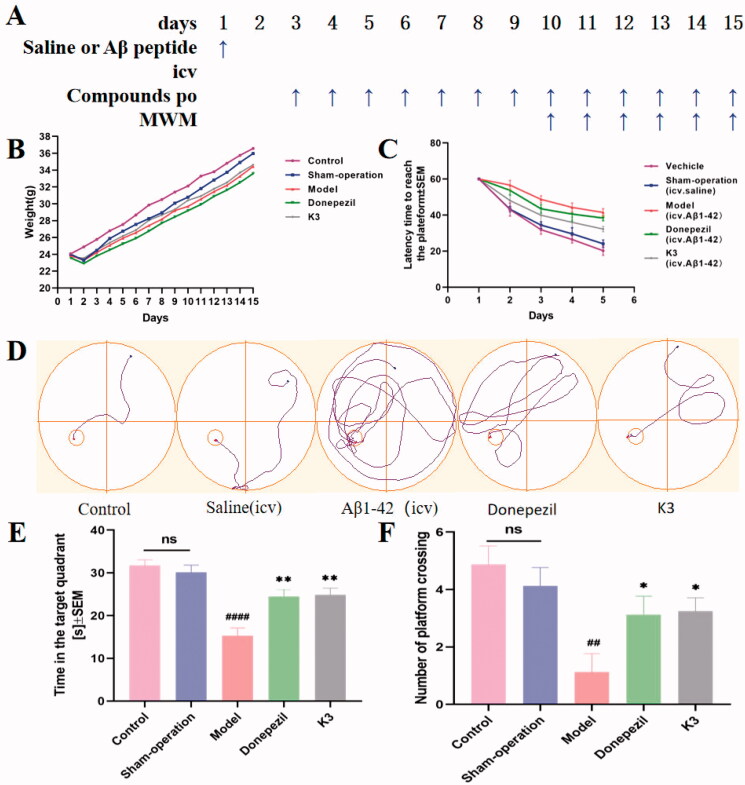
Effects of **K3** and donepezil on oligomeric A*β*_1–42_-induced damage experiments in the MWM task. (A) Protocol followed for *in vivo* experiments. (B) Daily body weight of mice in different groups during treatment. (C) Learning curves of the escape latencies during the acquisition phase of different groups. (D) Average footprints of mice in MWM on the last day of the study. (E) The time in the target quadrant during the acquisition phase of different groups. (F) The number of times the platform was crossed during the acquisition phase of different groups. icv: intraventricular injection; po: orally; MWM: Morris water maze. Data are presented as mean ± SEM (*n* = 8; ^##^*p* < 0.01, ^####^*p* < 0.0001 (vs control group), *p < 0.05, **p < 0.01 vs A*β*_1–42_ peptide model group).

We established a model of cognitive impairment generated by Aβ1–42 (intracerebroventricular (icv) injection) to explore compound **K3**'s anti-AD efficacy^51–53^. On day 1, amyloid peptide (10 µg per mouse) was injected into the ventricles of 30 mice, while a sham group was established, with the same amount of saline put into the ventricles. From Day 3 to Day 14, donepezil (15 mg/kg, as positive groups) and **K3** (10 mg/kg) were administered (po). Throughout the administration period, the animal's health and weight were monitored on a daily basis (Figure 9(B)). Compound **K3** had no effect on body weight increase, and there was no significant difference from the control group, indicating that **K3** is quite safe. From Day 10 to Day 14, behavioural studies were conducted. The Morris water maze test (MWM) was used. MWM was a spatial learning test that used distal cues to navigate from the starting point around the open swimming field to the underwater escape platform. MWM was primarily employed to investigate the impact of reducing the time it took to reach the escape platform (also known as escape time latency) on long-term memory^54^. MWM test included learning behaviour test on Days 10–14 and probe test on Day 15. Intraventricular injection of normal saline had no effect on the cognitive and learning abilities of mice, and there was no difference compared to the blank group in terms of the undifferentiated alternating behaviour, latency to reach the target, or confusion, as shown in Figure 9(C). The mice in the model group had much lower learning and memory abilities than those in the control group. As seen in Figure 9(D–F), the donepezil group was able to dramatically reduce the time it took to discover the platform and enhance the time spent on it when compared to the model group. Compared with the donepezil group, **K3** shortened the latency, simplified the movement trajectory to the platform, improved the interaction ability, and the overall target quadrant preference (the number of crossing the platform and the swimming time in the target quadrant), indicating that both **K3** (10 mg/kg) and donepezil (15 mg/kg) significantly improved the Aβ1–42-induced cognitive dysfunction.

**Figure 10.**
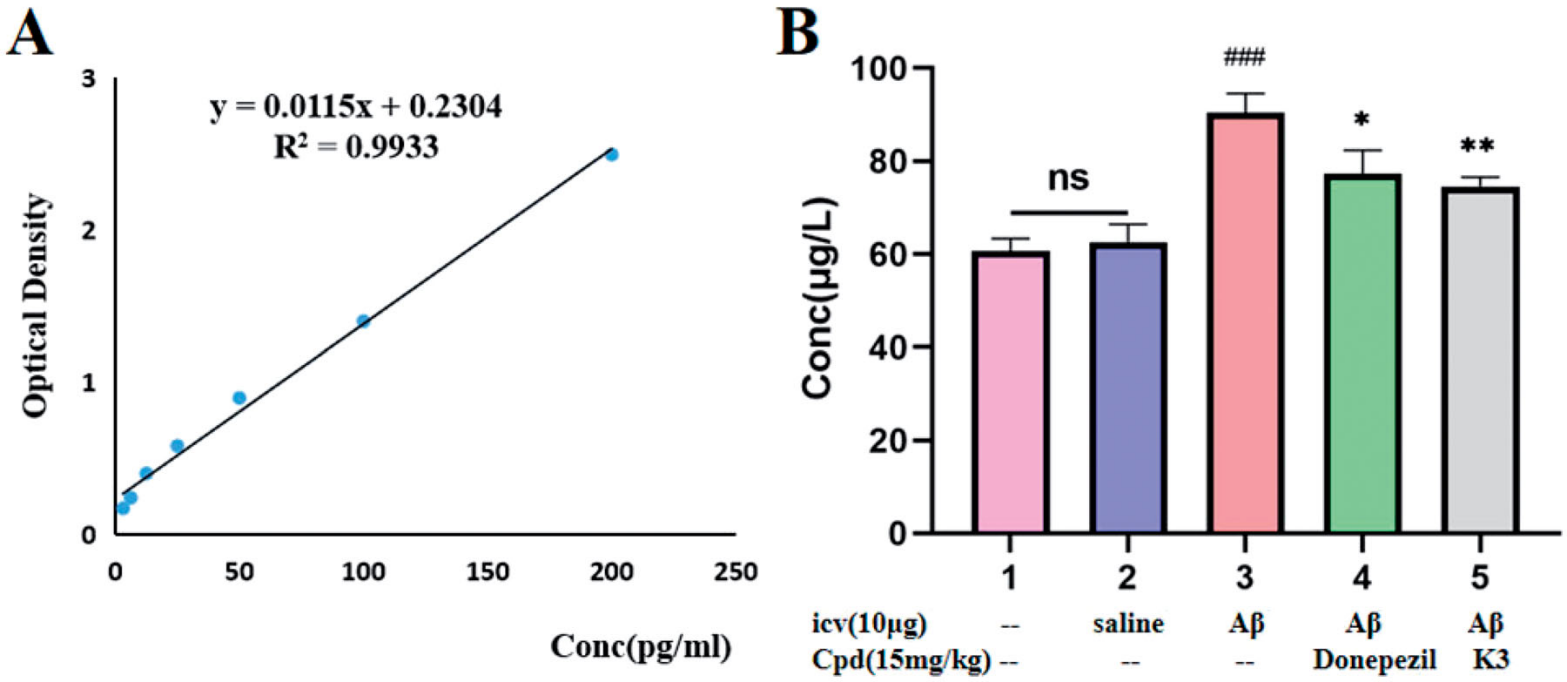
The A*β*_1 − 42_ total amount was quantifified by using a mouse A*β*_1 − 42_ ELISA kit. (A) Standard curve; (B) A*β*_1 − 42_ total amount in mice brains of different groups. Calculate brain tissue A*β*_1–42_ content according to linear regression equation, data are presented as mean ± SEM (*n* = 8; ^###^*p* < 0.001 (vs control group), *p < 0.05, **p < 0.01 vs Aβ1-42 peptide model group).

The mice were slaughtered at the end of the behavioural trial, and Aβ1–42 levels were evaluated using a mouse A*β*_1–42_ ELISA kit. As shown in Figure 10(A,B), the total levels of A*β*_1–42_ peptides in the icv A*β*_1–42_ group were significantly higher than the control or sham groups, indicating that the modelling was successful, and the A*β*_1–42_ peptides in the mice treated with donepezil or **K3** were significantly decreased (14.5% and 17.6%, respectively), consistent with the results of behavioural experiments, indicating that compound **K3** can further exert a neuroprotective effect on A*β*_1–42_ toxicity by reducing BuChE levels, thereby effectively improving the cognitive function of AD mice.

## Conclusion

A series of pyrazole-5-fluorosulfate derivatives were screened as selective BuChE inhibitors except for compound **K19**, amongst them, compounds **K3** showed potent BuChE inhibitory activity (IC_50_ = 0.79 μM). The structure-activity relationship (SAR) for pyrazole-5-fluorosulfates showed that the substituent at 1-phenyl ring affects the BuChE inhibitory activity: (i) 4-Cl > 4-F > 4-Br > 4-H > 4-CH_3_, such as **K3 **>** K2 **>** K1 **>** K4** for 3-phenyl, **K15 **>** K14 **>** K16 **>** K13** for 3-methyl, **K21 **>** K20 **>** K22** for 3-isopropyl; (ii) 3,4-diMe > mono-Me: **K5 **>** K4** for 3-phenyl, **K18 **>** K17** for 3-methyl, **K23 **>** K22** for 3-isopropyl; (iii) substituted *t*-Bu decreased BuChE inhibition, such as **K1 **>** K9**, **K13 **>** K19**; the order of 3-substituted pyrazole: (iv) –phenyl > –CH_3_ > –*i*-Pr, such as **K1 **>** K13 **>** K20** for 1-phenyl, **K2 **>** K14 **>** K21** for 4-F-phen-1-yl, **K3 **>** K15** for 4-Cl-phen-1-yl, **K4 **>** K22** for 4-Me-phen-1-yl, **K5 **>** K18 **>** K23** for 3,4-diMe-phen-1-yl, **K9 **>** K19** for 1-*t*-butyl. Molecular docking showed that compound **K3** could nicely insert into the binding groove of *h*BuChE(IC_50_ = 6.59 μM for hBuChE), forming π-π, π-S and halogen bond multiple interactions. According to kinetic studies, compound **K3** (Ki = 0.77 M) was a reversible, mixed, and non-competitive BuChE inhibitor. Additionally, compound **K3** demonstrated exceptional neuroprotective efficacy and moderate BBB penetration potential. Compound **K3** had good neurological and hepatic safety and was tolerated up to a dose of 1.0 g/kg, according to *in vitro* and in vivo safety experiments. In a following in vivo behavioural research, **K3** therapy increased the A*β*_1 − 42_-induced cognitive impairment, greatly reduced A*β*_1 − 42_ toxicity, and nearly recovered cognitive function. Furthermore, the evaluation of the A*β*_1 − 42_ total amount confirmed its anti-amyloidogenic effects better than the positive donepezil. As a result, compound **K3** has the potential to be further developed into an effective therapy for the treatment of AD.

The data of ChE inhibition assays that were used to calculate the IC_50_ and enzyme kinetics, the copies of representative ^1^H and ^13^C NMR spectra and the copies of HPLC spectra can be found in Supplementary materials.

## Supplementary Material

Supplemental Material
