# Supplementary material for: Fluorosulfate-containing pyrazole heterocycles as selective BuChE inhibitors: structure-activity relationship and biological evaluation for the treatment of Alzheimer’s disease
Source: J Enzyme Inhib Med Chem. 2022 Jul 28;37(1):2099–111. doi: 10.1080/14756366.2022.2103553 (PMC9448382; doi:10.1080/14756366.2022.2103553)
Supplement: Supplemental Material [file IENZ_A_2103553_SM7555.pdf]

Fluorosulfate-containing pyrazole heterocycles as selective BuChE inhibitors: structure-activity relationship and biological evaluation for the treatment of Alzheimer's disease

Huan-Huan Li [a,1], Chengyao Wu [a,1], Shi-Long Zhang [a], Jian-Guo Yang [a], Hua-Li Qin [b,*], Wenjian Tang [a,*]

[a] *School of Pharmacy, Anhui Medical University, Hefei 230032, China*

[b] *School of Chemistry, Chemical Engineering and Life Science, Wuhan University of Technology, Wuhan 430070, China*

Content

Molecular docking of compounds M3 and K3……………………...………………**S2**

General procedure for the synthesis of compounds K1–K26 .... **S3-S10**

Copies of HPLC of compounds K1–K26 .... **S11-S36**

Molecular docking of compounds ***M3*** *and* ***K3***

To better understand the capacity of compounds **M3** and **K3** targeting hBuChE, their binding modes were investigated by the CDOCKER molecular docking module in Discovery Studio 2018. As shown in Fig. S1, compound **K3** could nicely insert into the binding groove of hBuChE, forming multiple π-π interactions between the benzene ring and Gly116 and Trp231 and Phe329, between the chlorine and Asn83. The fluorosulfate of the pyrazole ring of compound **M3** increased the inhibitory activity of BuChE through π-sulfur interaction between the sulfur and Trp82. Therefore, 1,3-disubstituted pyrazole-5-sulfofluoridates could be used to carry out ChE inhibitory screening.

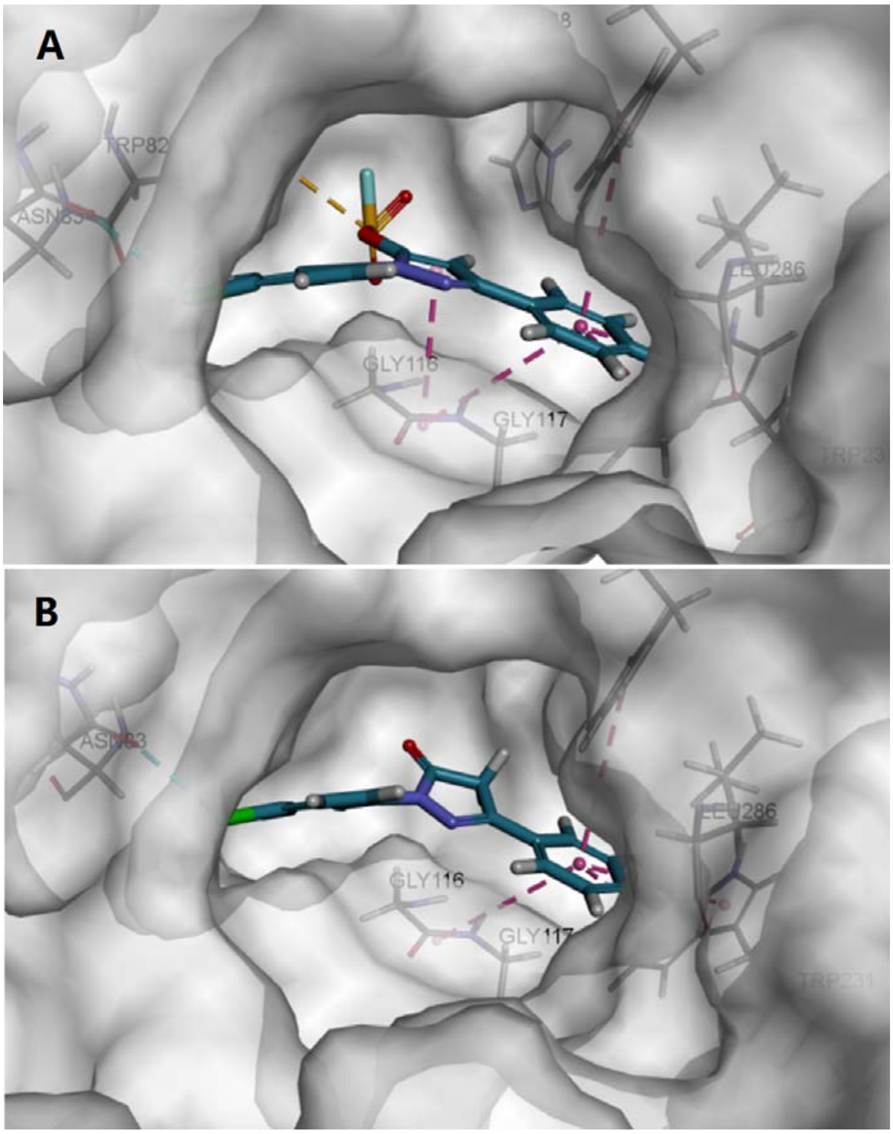

Fig. S1. 3D diagram of compounds **K3** (**A**) and **M3** (**B**) in human BuChE (PDB code 6QAA).

Synthesis of pyrazole-5-fluorosulfate derivatives ***K1–K26****.*

General information

Recently, we constructed a class of novel *N*-heterocyclic molecules containing both pyrazole and fluorosulfate as versatile building blocks in the Suzuki coupling reaction and SuFEx click chemistry. Synthetic procedures and characterization of compounds **K1–K26** were reported in the literature. The purity (relative content) of active compounds was determined by HPLC on an Agilent 1200 instrument (column: Elite, RP-C18, 5 μm, 4.6 × 150 mm) through area normalization method. TLC was carried out on pre-coated silica gel F_{254} glass plates with petroleum ether/ethyl acetate (10: 1 → 4: 1).

General procedure for the synthesis of compounds ***K1–K26****.*

An oven-dried reaction tube (20 mL) was charged with pyrazolone **A** (1.0 mmol), DIPEA (1.5 mmol), 5 mL CH_2Cl_2 and a SO_2F_2 balloon. The mixture was stirred at room temperature for 5–10 h with monitoring by TLC. After the reaction was completed, the solution was concentrated to dryness and the residue was purified by flash silica gel column chromatography using a mixture of ethyl acetate and petroleum ether to afford the desired products **K1–K26**.

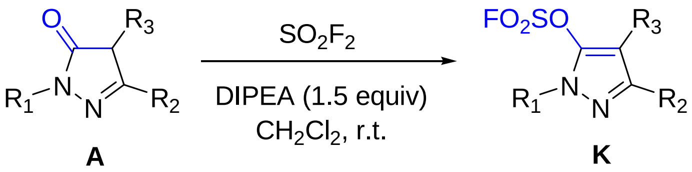

*1,3-Diphenyl-1H-pyrazol-5-yl sulfurofluoridate (**K1**).* White solid, 259 mg, 81%; mp 56–57 °C; purity, 99.9%; R_f = 0.48 in petroleum ether/ethyl acetate (10: 1); ^{1}H NMR (500 MHz, $CDCl_3$) δ 7.86 (d, J = 7.1 Hz, 2H), 7.64 (d, J = 7.5 Hz, 2H), 7.54 (t, J = 7.8 Hz, 2H), 7.45 (t, J = 7.4 Hz, 3H), 7.39 (t, J = 7.3 Hz, 1H), 6.73 (s, 1H); ^{13}C NMR (126 MHz, $CDCl_3$) δ 151.4 (s), 142.0 (s), 136.9 (s), 132.1 (s), 129.7 (s), 129.1 (s), 128.9 (s), 128.8 (s), 125.7 (s), 123.7 (s), 93.9 (s); ^{19}F NMR (471 MHz, $CDCl_3$) δ 39.9 (s, 1F); ESI-MS HRMS calculated for $C_{15}H_{12}FN_2O_3S$ $[M + H]^+$ 319.0547, found 319.0545.

*1-(4-Fluorophenyl)-3-phenyl-1H-pyrazol-5-yl sulfurofluoridate (**K2**).* White solid,

246 mg, 73%, mp 59–61 °C; purity, 99.7%; R_f = 0.48 in petroleum ether/ethyl acetate (10: 1); ^{1}H NMR (500 MHz, $CDCl_3$) δ 7.84 (d, J = 7.3 Hz, 2H), 7.62–7.59 (m, 2H), 7.45 (t, J = 7.4 Hz, 2H), 7.39 (t, J = 7.3 Hz, 1H), 7.23 (t, J = 8.5 Hz, 2H), 6.72 (s, 1H); ^{13}C NMR (126 MHz, $CDCl_3$) δ 162.5 (d, J = 249.4 Hz), 151.5 (s), 142.0 (s), 133.0 (d, J = 3.1 Hz), 131.9 (s), 129.2 (s), 129.0 (s), 125.8 (d, J = 8.8 Hz), 125.7 (s), 116.7 (d, J = 23.2 Hz), 93.9 (s); ^{19}F NMR (471 MHz, $CDCl_3$) δ 39.9 (s, 1F), −111.86 to −111.92 (m, 1F); ESI-MS HRMS calculated for $C_{15}H_{11}F_2N_2O_3S$ $[M+ H]^+$ 337.0453, found 337.0452.

*1-(4-Chlorophenyl)-3-phenyl-1H-pyrazol-5-yl sulfurofluoridate (**K3**)*. Yellow liquid, 234 mg, 66%, mp 50–51 °C; purity, 96.5%; R_f = 0.55 in petroleum ethe /ethyl acetate (10: 1);^{1}H NMR (500 MHz, $CDCl_3$) δ 7.84 (d, J = 8.3, 2H), 7.60 (d, J = 8.6 Hz, 2H), 7.51 (d, J = 8.9 Hz, 2H), 7.45 (t, J = 7.98 Hz, 2H), 7.40 (t, J = 7.9 Hz, 1H), 6.73 (s, 1H); ^{13}C NMR (126 MHz, $CDCl_3$) δ 151.7 (s), 141.9 (s), 135.4 (s), 134.6 (s), 131.8 (s), 129.9 (s), 129.2 (s), 129.0 (s), 125.7 (s), 124.7 (s), 94.2 (s); ^{19}F NMR (471 MHz, $CDCl_3$) δ 40.1 (s, 1F); ESI-MS HRMS calculated for $C_{15}H_{11}ClFN_2O_3S$ $[M + H]^+$ 353.0157, found 353.0157.

*3-Phenyl-1-(p-tolyl)-1H-pyrazol-5-yl sulfurofluoridate (**K4**)*. White solid, 264 mg, 80%, mp 84–85 °C; purity, 98.8%; Rf = 0.52 in petroleum ether/ethyl acetate (10: 1);1H NMR (500 MHz, CDCl3) δ 7.85 (d, J = 8.4 Hz, 2H), 7.50 (d, J = 8.3 Hz, 2H), 7.44 (t, J = 7.4 Hz, 2H), 7.38 (t, J = 7.3 Hz, 1H), 7.33 (d, J = 8.2 Hz, 2H), 6.71 (s, 1H), 2.44 (s, 3H); 13C NMR (126 MHz, CDCl3) δ 151.1 (s), 141.9 (s), 139.0 (s), 134.4 (s), 132.2 (s), 130.2 (s), 129.0 (s), 128.9 (s), 125.7 (s), 123.7 (s), 93.6 (s), 21.3 (s); 19F NMR (471 MHz, CDCl3) δ 39.9 (s, 1F); ESI-MS HRMS calculated for C16H14FN2O3S [M + H]+ 333.0704, found 333.0703.

*1-(3,4-Dimethylphenyl)-3-phenyl-1H-pyrazol-5-yl sulfurofluoridate (**K5**)*. Yellow solid, 346 mg, quant, mp 58–59 °C; purity, 99.9%; R_f = 0.48 in petroleum ether/ethyl acetate (10 : 1); ^{1}H NMR (500 MHz, $CDCl_3$) δ 7.85 (d, J = 8.5 Hz, 2H), 7.44 (t, J = 7.4 Hz, 2H), 7.40–7.36 (m, 2H), 7.32–7.30 (m, 1H), 7.27 (d, J = 7.2 Hz, 1H), 6.70 (s, 1H), 2.35 (s, 3H) 2.33 (s, 3H); ^{13}C NMR (126 MHz, $CDCl_3$) δ 151.0 (s), 141.9 (s), 138.3 (s), 137.7 (s), 134.5 (s), 132.2 (s), 130.6 (s), 128.93 (s), 128.90 (s), 125.7 (s), 125.0 (s), 121.1 (s), 93.5 (s), 20.0 (s), 19.7 (s); ^{19}F NMR (471

MHz, $CDCl_3$) δ 39.9 (s, 1F); ESI-MS HRMS calculated for $C_{17}H_{16}FN_2O_3S$ $[M + H]^+$ 347.0860, found 347.0859.

*1-(4-Methoxyphenyl)-3-phenyl-1H-pyrazol-5-yl sulfurofluoridate (**K6**).* White solid, 260 mg, 75%, mp 79–80 °C; purity, 99.9%; R_f = 0.50 in petroleum ether/ethyl acetate (6 : 1); ^{1}H NMR (500 MHz, $CDCl_3$) δ 7.85 (d, J = 7.1 Hz, 2H), 7.52 (d, J = 9.0 Hz, 2H), 7.44 (t, J = 7.4 Hz, 2H), 7.38 (t, J = 7.3 Hz, 1H), 7.03 (d, J = 9.0 Hz, 2H), 6.70 (s, 1H), 3.87 (s, 3H); ^{13}C NMR (126 MHz, $CDCl_3$) δ 159.9 (s), 151.0 (s), 141.9 (s), 132.2 (s), 129.8 (s), 128.92 (s), 128.89 (s), 125.7 (s), 125.5 (s), 114.8 (s), 93.4 (s), 55.7 (s); ^{19}F NMR (471 MHz, $CDCl_3$) δ 39.8 (s, 1F); ESI-MS HRMS calculated for $C_{16}H_{14}FN_2O_4S$ $[M + H]^+$ 349.0653, found 349.0651.

*3-Phenyl-1-(pyridin-2-yl)-1H-pyrazol-5-yl sulfurofluoridate (**K7**).* White solid, 172 mg, 54%, mp 80–81 °C; purity, 99.1%; R_f = 0.50 in petroleum ether/ethyl acetate (6 : 1); ^{1}H NMR (500 MHz, $CDCl_3$) δ 8.53 (d, J = 4.6 Hz, 1H), 7.98 (d, J = 8.2 Hz, 1H), 7.90–7.87 (m, 3H), 7.46 (t, J = 7.4 Hz, 2H), 7.41 (t, J = 7.3 Hz, 1H), 7.30 (t, J = 6.1 Hz, 1H), 6.74 (s, 1H); ^{13}C NMR (126 MHz, $CDCl_3$) δ 151.4 (s), 151.3 (s), 148.3 (s), 142.7 (s), 138.9 (s), 131.8 (s), 129.3 (s), 129.0 (s), 125.9 (s), 122.6 (s), 115.4 (s), 96.4 (s); ^{19}F NMR (471 MHz, $CDCl_3$) δ 42.3 (s, 1F); ESI-MS HRMS calculated for $C_{14}H_{11}FN_3O_3S$ $[M + H]^+$ 320.0500, found 320.0457.

*1-Cyclohexyl-3-phenyl-1H-pyrazol-5-yl sulfurofluoridate (**K8**).* Yellow liquid, 324 mg, quant; purity, 99.3%; R_f = 0.58 in petroleum ether/ethyl acetate (10: 1);^{1}H NMR (500 MHz, $CDCl_3$) δ 7.81 (d, J = 7.5 Hz, 2H), 7.43 (t, J = 7.6 Hz, 2H), 7.36 (t, J = 7.3 Hz, 1H), 6.51 (s, 1H), 4.17 (m, 1H), 2.08–2.02 (m, 4H), 1.98–1.95 (m, 2H), 1.78–1.76 (m, 1H), 1.51–1.42 (m, 2H), 1.38–1.32 (m, 1H); ^{13}C NMR (126 MHz, $CDCl_3$) δ 149.8 (s), 141.1 (s), 132.9 (s), 128.7 (s), 128.4 (s), 125.4 (s), 91.5 (s), 57.9 (s), 32.5 (s), 25.5 (s), 25.1 (s); ^{19}F NMR (471 MHz, $CDCl_3$) δ 37.8 (s, 1F); ESI-MS HRMS calculated for $C_{15}H_{18}FN_2O_3S$ $[M + H]^+$ 325.1017, found 325.1014.

*1-(tert-Butyl)-3-phenyl-1H-pyrazol-5-yl sulfurofluoridate (**K9**).* Yellow liquid, 298 mg, quant;purity, 99.8%; R_f = 0.58 in petroleum ether/ethyl acetate (12: 1); ^{1}H NMR (500 MHz, $CDCl_3$) δ 7.78 (d, J = 7.6 Hz, 2H), 7.40 (t, J = 7.6 Hz, 2H), 7.33 (t, J = 7.3Hz, 1H), 6.53 (s, 1H), 1.70 (s, 9H); ^{13}C NMR (126 MHz, $CDCl_3$) δ 148.0 (s), 142.3

(s), 132.8 (s), 128.7 (s), 128.3 (s), 125.3 (s), 92.4 (s), 61.4 (s), 29.4 (s); ^{19}F NMR (471 MHz, $CDCl_3$) δ 39.7 (s, 1F); ESI-MS HRMS calculated for $C_{13}H_{16}FN_2O_3S$ $[M + H]^+$ 299.0860, found 299.0860.

*3-Phenyl-1H-pyrazol-5-yl sulfurofluoridate (**K10**).* White solid, 227 mg, mp 129–120 °C; purity, 99.9%; R_f = 0.29 in petroleum ether/ethyl acetate (4 : 1); ^{1}H NMR (500 MHz, $CDCl_3$) δ 11.33 (s, 1H), 7.58 (d, J = 7.1 Hz, 2H), 7.50–7.45 (m, 3H), 6.45 (s, 1H); ^{13}C NMR (126 MHz, $CDCl_3$) δ 154.2 (s), 145.9 (s), 130.1 (s), 129.5 (s), 127.9 (s), 125.7 (s), 92.8 (s); ^{19}F NMR (471 MHz, $CDCl_3$) δ 39.2 (s, 1F); ESI-MS HRMS calculated for $C_9H_8FN_2O_3S$ $[M + H]^+$ 243.0234, found 243.0234.

*3,4-Dimethyl-1-phenyl-1H-pyrazol-5-yl sulfurofluoridate (**K11**).* Colorless liquid, 270 mg, quant; purity, 99.6%; R_f = 0.54 in petroleum ether/ethyl acetate (6 : 1); ^{1}H NMR (500 MHz, $CDCl_3$) δ 7.52–7.46 (m, 4H), 7.37 (t, J = 7.2 Hz, 1H), 2.28 (s, 3H), 2.07 (s, 3H); ^{13}C NMR (126 MHz, $CDCl_3$) δ 148.9 (s), 138.7 (s), 137.3 (s), 129.6 (s), 128.1 (s), 123.2 (s), 105.7 (s), 12.9 (s), 7.0 (d, J = 1.6 Hz); ^{19}F NMR (471 MHz, $CDCl_3$) δ 42.0 (s, 1F); ESI-MS HRMS calculated for $C_{11}H_{12}FN_2O_3S$ $[M + H]^+$ 271.0547, found 271.0546.

*3,4-Dimethyl-1-(pyridin-2-yl)-1H-pyrazol-5-yl sulfurofluoridate (**K12**).* Yellow liquid, 60 mg, 22%; purity, 99.6%; R_f = 0.41 in petroleum ether/ethyl acetate (6 : 1); ^{1}H NMR (500 MHz, $CDCl_3$) δ 8.45 (d, J = 4.6 Hz, 1H), 7.81–7.77 (m, 2H), 7.20–7.18 (m, 1H), 2.27 (s, 3H), 2.06 (s, 3H); ^{13}C NMR (126 MHz, $CDCl_3$) δ 151.4 (s), 149.5 (s), 148.0 (s), 139.5 (s), 138.7 (s), 121.8 (s), 114.3 (s), 107.6 (s), 13.0 (s), 6.7 (s); ^{19}F NMR (471 MHz, $CDCl_3$) δ 46.1 (s, 1F); ESI-MS HRMS calculated for $C_{10}H_{11}FN_3O_3S$ $[M + H]^+$ 272.0500, found 272.0500.

*3-Methyl-1-phenyl-1H-pyrazol-5-yl sulfurofluoridate (**K13**).* Colorless liquid, 238 mg, 93%; purity, 98.9%; R_f = 0.52 in petroleum ether/ethyl acetate (6 : 1); ^{1}H NMR (500 MHz, $CDCl_3$) δ 7.53–7.47 (m, 4H), 7.40 (t, J = 7.2 Hz, 1H), 6.22 (s, 1H), 2.35 (s, 3H); ^{13}C NMR (126 MHz, $CDCl_3$) δ 149.4 (s), 141.3 (s), 136.8 (s), 129.6 (s), 128.5 (s), 123.5 (s), 96.2 (s), 14.7 (s); ^{19}F NMR (471 MHz, $CDCl_3$) δ 39.6 (s, 1F); ESI-MS HRMS calculated for $C_{10}H_{10}FN_2O_3S$ $[M + H]^+$ 257.0391, found 257.0390.

*1-(4-Fluorophenyl)-3-methyl-1H-pyrazol-5-yl sulfurofluoridate (**K14**)*. Yellow liquid, 219 mg, 80%; purity, 96.3%; R_f = 0.52 in petroleum ether/ethyl acetate (6 : 1); ^{1}H NMR (500 MHz, $CDCl_3$) δ 7.51–7.48 (m, 2H), 7.20–7.16 (m, 2H), 6.21 (s, 1H), 2.33 (s, 3H); ^{13}C NMR (126 MHz, $CDCl_3$) δ 162.3 (d, J = 249.0 Hz), 149.5 (s), 141.3 (s), 132.9 (d, J = 3.1 Hz), 125.6 (d, J = 8.7 Hz), 116.6 (d, J = 23.1 Hz), 96.2 (s), 14.6 (s); ^{19}F NMR (471 MHz, $CDCl_3$) δ 39.5 (s, 1F), −112.38 to −112.43 (m, 1F); ESI-MS HRMS calculated for $C_{10}H_9F_2N_2O_3S$ $[M + H]^+$ 275.0296, found 275.0295.

*1-(4-Chlorophenyl)-3-methyl-1H-pyrazol-5-yl sulfurofluoridate (**K15**)*. Yellow liquid, 162 mg, 56%; purity, 98.9%; R_f = 0.55 in petroleum ether/ethyl acetate (6 : 1); ^{1}H NMR (500 MHz, $CDCl_3$) δ 7.49–7.45 (m, 4H), 6.22 (s, 1H), 2.33 (s, 3H); ^{13}C NMR (126 MHz, $CDCl_3$) δ 149.7 (s), 141.3 (s), 135.3 (s), 134.2 (s), 129.8 (s), 124.5 (s), 96.5 (s), 14.6 (s); ^{19}F NMR (471 MHz, $CDCl_3$) δ 39.7 (s, 1F); ESI-MS HRMS calculated for $C_{10}H_9ClFN_2O_3S$ $[M+ H]^+$ 291.0001, found 291.0001.

*1-(4-Bromophenyl)-3-methyl-1H-pyrazol-5-yl sulfurofluoridate (**K16**)*. Colorless liquid, 281 mg, 84%; purity, 99.7%; R_f = 0.50 in petroleum ether/ethyl acetate (6 : 1); ^{1}H NMR (500 MHz,$CDCl_3$) δ 7.62 (d, J = 8.9 Hz, 2H), 7.42 (d, J = 8.9 Hz, 2H), 6.22 (s, 1H), 2.34 (s, 3H); ^{13}C NMR (126 MHz, $CDCl_3$) δ 149.8 (s), 141.3 (s), 135.9 (s), 132.8 (s), 124.7 (s), 122.1 (s), 96.6 (s), 14.7 (s); ^{19}F NMR (471 MHz, $CDCl_3$) δ 39.7 (s, 1F); ESI-MS HRMS calculated for $C_{10}H_9BrFN_2O_3S$ $[M + H]^+$ 334.9496, found 334.9494.

*3-Methyl-1-(m-tolyl)-1H-pyrazol-5-yl sulfurofluoridate (**K17**).* Colorless liquid, 194 mg, 72%; purity, 99.7%; R_f = 0.55 in petroleum ether/ethyl acetate (12: 1); ^{1}H NMR (500 MHz, $CDCl_3$) δ 7.37–7.34 (m, 2H), 7.30–7.29 (m, 1H), 7.22–7.20 (m, 1H), 6.20 (s, 1H), 2.41 (s, 3H), 2.34 (s, 3H); ^{13}C NMR (126 MHz, $CDCl_3$) δ 149.2 (s), 141.2 (s), 139.8 (s), 136.7 (s), 129.28 (s), 129.27 (s), 124.2 (s), 120.4 (s), 96.0 (s), 21.5 (s), 14.6 (s); ^{19}F NMR (471 MHz, $CDCl_3$) δ 39.6 (s, 1F); ESI-MS HRMS calculated for $C_{11}H_{12}FN_2O_3S$ $[M + H]^+$ 271.0547, found 271.0543.

*1-(3,4-Dimethylphenyl)-3-methyl-1H-pyrazol-5-yl sulfurofluoridate (**K18**)*. Yellow liquid, 217 mg, 76%; purity, 99.3%; R_f = 0.50 in petroleum ether/ethyl acetate (6 : 1); ^{1}H NMR (500 MHz, $CDCl_3$) δ 7.30 (s, 1H), 7.21 (m, 2H), 6.18 (s, 1H), 2.34 (s, 3H),

2.31 (s, 3H), 2.30 (s, 3H); ^{13}C NMR (126 MHz, $CDCl_3$) δ 149.0 (s), 141.2 (s), 138.2 (s), 137.3 (s), 134.5 (s), 130.5 (s), 124.8 (s), 120.8 (s), 95.8 (s), 19.9 (s), 19.6 (s), 14.6 (s); ^{19}F NMR (471 MHz, $CDCl_3$) δ 39.5 (s, 1F); ESI-MS HRMS calculated for $C_{12}H_{14}FN_2O_3S$ $[M + H]^+$ 285.0704, found 285.0704.

*1-(tert-Butyl)-3-methyl-1H-pyrazol-5-yl sulfurofluoridate (**K19**)*. Yellow liquid, 236 mg, quant; purity, 99.7%; R_f = 0.29 in petroleum ether/ethyl acetate (4 : 1); ^1H NMR (500 MHz, $CDCl_3$) δ 6.00 (s, 1H), 2.22 (s, 3H), 1.60 (s, 9H);^{13}C NMR (126 MHz, $CDCl_3$) δ 145.9 (s), 141.6 (s), 94.7 (s), 60.6 (s), 29.4 (s), 14.5 (s); ^{19}F NMR (471 MHz, $CDCl_3$) δ 39.3 (s, 1F); ESI-MS HRMS calculated for $C_8H_{14}FN_2O_3S$ $[M+ H]^+$ 237.0704, found 237.0702.

*3-Isopropyl-1-phenyl-1H-pyrazol-5-yl sulfurofluoridate (**K20**)*. Yellow liquid, 233 mg, 82%; purity, 99.7%; R_f = 0.55 in petroleum ether/ethyl acetate (12: 1); ^1H NMR (500 MHz, $CDCl_3$) δ 7.55 (d, J = 7.6 Hz, 2H), 7.49 (t, J = 7.9 Hz, 2H), 7.40 (t, J = 7.3 Hz, 1H), 6.24 (s, 1H), 3.02 (hept, J = 6.9 Hz, 1H), 1.32 (d, J = 7.0 Hz, 6H); ^{13}C NMR (126 MHz, $CDCl_3$) δ 159.4 (s), 141.1 (s), 137.0 (s), 129.6 (s), 128.4 (s), 123.6 (s), 93.5 (s), 28.8 (s), 22.4 (s); ^{19}F NMR (471 MHz, $CDCl_3$) δ 39.5 (s, 1F); ESI-MS HRMS calculated for $C_{12}H_{14}FN_2O_3S$ $[M + H]^+$ 285.0704, found 285.0701.

*1-(4-Fluorophenyl)-3-isopropyl-1H-pyrazol-5-yl sulfurofluoridate (**K21**)*. Green liquid, 252 mg, 83%; purity, 99.8%; R_f = 0.43 in petroleum ether/ethyl acetate (10: 1); ^1H NMR (500 MHz, $CDCl_3$) δ 7.53–7.50 (m, 2H), 7.20–7.16 (m, 2H), 6.23 (s, 1H), 3.00 (hept, J = 6.9 Hz, 1H), 1.30 (d, J = 7.0 Hz, 6H); ^{13}C NMR (126 MHz, $CDCl_3$) δ 159.2 (s), 141.0 (s), 138.5 (s), 134.5 (s), 130.1 (s), 123.5 (s), 93.2 (s), 28.8 (s), 22.4 (s), 21.2 (s); ^{19}F NMR (471 MHz, $CDCl_3$) δ 39.5 (s, 1F), −112.57 to −112.62 (m, 1F); ESI-MS HRMS calculated for $C_{12}H_{13}F_2N_2O_3S$ $[M + H]^+$ 303.0609, found 303.0608.

*3-Isopropyl-1-(p-tolyl)-1H-pyrazol-5-yl sulfurofluoridate (**K22**)*. Yellow liquid, 257 mg, 86%; purity, 99.2%; R_f = 0.43 in petroleum ether/ethyl acetate (10: 1); ^1H NMR (500 MHz, $CDCl_3$) δ 7.41 (d, J = 8.4 Hz, 2H), 7.28 (d, J = 8.2 Hz, 2H), 6.22 (s, 1H), 3.01 (hept, J = 7.0 Hz, 1H), 2.40 (s, 3H), 1.31 (d, J = 7.0 Hz, 6H);^{13}C NMR (126 MHz, $CDCl_3$) δ 163.2 (s), 161.3 (s), 159.5 (s), 141.1 (s), 133.0 (d, J = 3.1 Hz), 125.6 (d, J = 8.7 Hz), 116.6 (d, J = 23.1 Hz), 93.5 (s), 28.8 (s), 22.3 (s); ^{19}F NMR (471 MHz,

$CDCl_3$) δ 39.5 (s, 1F); ESI-MS HRMS calculated for $C_{13}H_{16}FN_2O_3S$ $[M + H]^+$ 299.0860, found 299.0860.

*1-(3,4-Dimethylphenyl)-3-isopropyl-1H-pyrazol-5-yl sulfurofluoridate (**K23**).* Yellow liquid, 312 mg, quant;purity, 99.0%; R_f = 0.46 in petroleum ether/ethyl acetate (10: 1); ^{1}H NMR (500 MHz, $CDCl_3$) δ 7.31 (s, 1H), 7.22 (s, 2H), 6.21 (s, 1H), 3.01 (m, 1H), 2.31 (s, 3H), 2.30 (s, 3H), 1.31 (d, J = 6.7 Hz, 6H); ^{13}C NMR (126 MHz, $CDCl_3$) δ 159.1 (s), 141.0 (s), 138.2 (s), 137.2 (s), 134.7 (s), 130.5 (s), 124.9 (s), 120.9 (s), 93.1 (s), 28.8 (s), 22.5 (s), 20.0 (s), 19.6 (s); ^{19}F NMR (471 MHz, $CDCl_3$) δ 39.5 (s, 1F); ESI-MS HRMS calculated for $C_{14}H_{18}FN_2O_3S$ $[M + H]^+$ 313.1017, found 313.1017.

*1-(4-Chlorophenyl)-3-propyl-1H-pyrazol-5-yl sulfurofluoridate (**K24**).* Yellow liquid, 238 mg, 75%; purity, 98.4%; R_f = 0.66 in petroleum ether/ethyl acetate (10: 1); ^{1}H NMR (500 MHz, $CDCl_3$) δ 7.50–7.45 (m, 4H), 6.23 (s, 1H), 2.63 (t, J = 7.5 Hz, 2H), 1.71 (m, 2H), 1.00 (t, J = 7.4 Hz, 3H); ^{13}C NMR (126 MHz, $CDCl_3$) δ 154.2 (s), 141.2 (s), 135.4 (s), 134.2 (s), 129.8 (s), 124.5 (s), 95.6 (s), 31.2 (s), 22.4 (s), 13.9 (s); ^{19}F NMR (471 MHz, $CDCl_3$) δ 39.7 (s, 1F); ESI-MS HRMS calculated for $C_{12}H_{13}ClFN_2O_3S$ $[M + H]^+$ 319.0314, found 319.0313.

*1-(4-Methoxyphenyl)-3-propyl-1H-pyrazol-5-yl sulfurofluoridate (**K25**).* Yellow liquid, 241 mg, 78%; purity, 99.9%; R_f = 0.38 in petroleum ether/ethyl acetate (10: 1); ^{1}H NMR (500 MHz, $CDCl_3$) δ 7.41 (d, J = 9.0 Hz, 2H), 6.98 (d, J = 9.0 Hz, 2H), 6.18 (s, 1H), 3.84 (s, 3H), 2.63 (t, J = 7.8 Hz, 2H), 1.71 (m, 2H), 0.99(t, J = 7.4 Hz, 3H); ^{13}C NMR (126 MHz, $CDCl_3$) δ 159.6 (s), 153.5 (s), 141.1 (s), 129.8 (s), 125.4 (s), 114.7 (s), 94.7 (s), 55.7 (s), 31.2 (s), 22.5 (s), 13.9 (s); ^{19}F NMR (471 MHz, $CDCl_3$) δ 39.4 (s, 1F); ESI-MS HRMS calculated for $C_{13}H_{16}FN_2O_4S$ $[M + H]^+$ 315.0809, found 315.0807.

*3-Cyclopropyl-1-phenyl-1H-pyrazol-5-yl sulfurofluoridate (**K26**).* Yellow liquid, 282 mg, quant; purity, 99.4%; R_f = 0.55 in petroleum ether/ethyl acetate (10: 1); ^{1}H NMR (500 MHz, $CDCl_3$) δ 7.53–7.47 (m, 4H), 7.39 (t, J = 7.2 Hz, 1H), 6.09 (s, 1H), 1.97 (m, 1H), 1.00–0.98 (m, 2H), 0.84–0.83 (m, 2H); ^{13}C NMR (126 MHz, $CDCl_3$) δ 155.6 (s), 141.1 (s), 136.9 (s), 129.6 (s), 128.4 (s), 123.5 (s), 93.4 (s), 10.0 (s), 8.2 (s);

^{19}F NMR (471 MHz, $CDCl_3$) δ 39.6 (s, 1F); ESI-MS HRMS calculated for $C_{12}H_{12}FN_2O_3S$ $[M + H]^+$ 283.0547, found 283.0546.

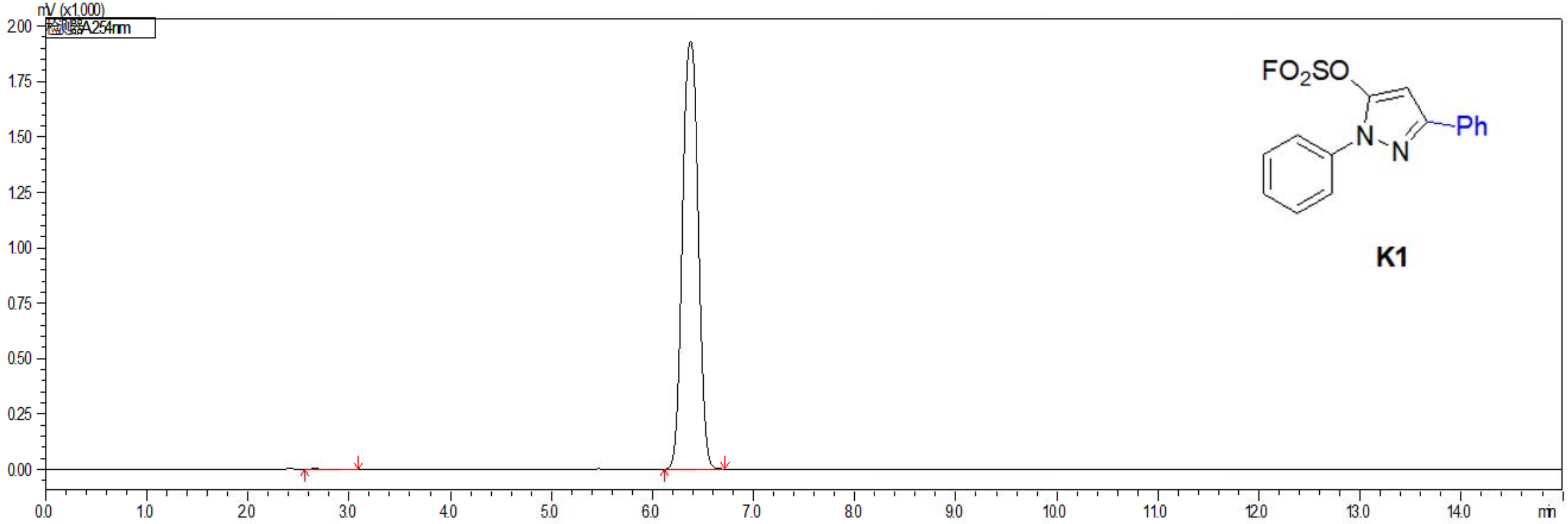

No	Ret Time(min)	Area(mAU*min)	Rel.Area(%)
1	2.658	24847	0.117
2	6.375	21233478	99.883
Total		21258325	

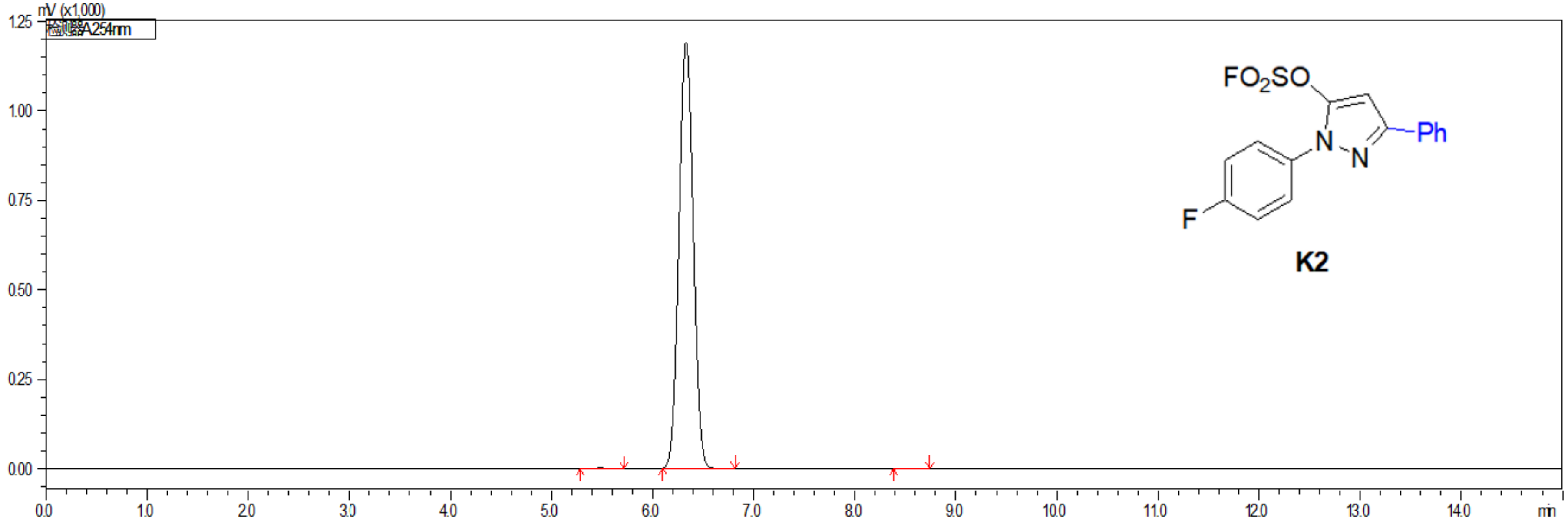

No	Ret Time(min)	Area(mAU*min)	Rel.Area(%)
1	5.489	18220	0.154
2	6.33	11765761	99.718
3	8.55	15077	0.128
Total		11799058	

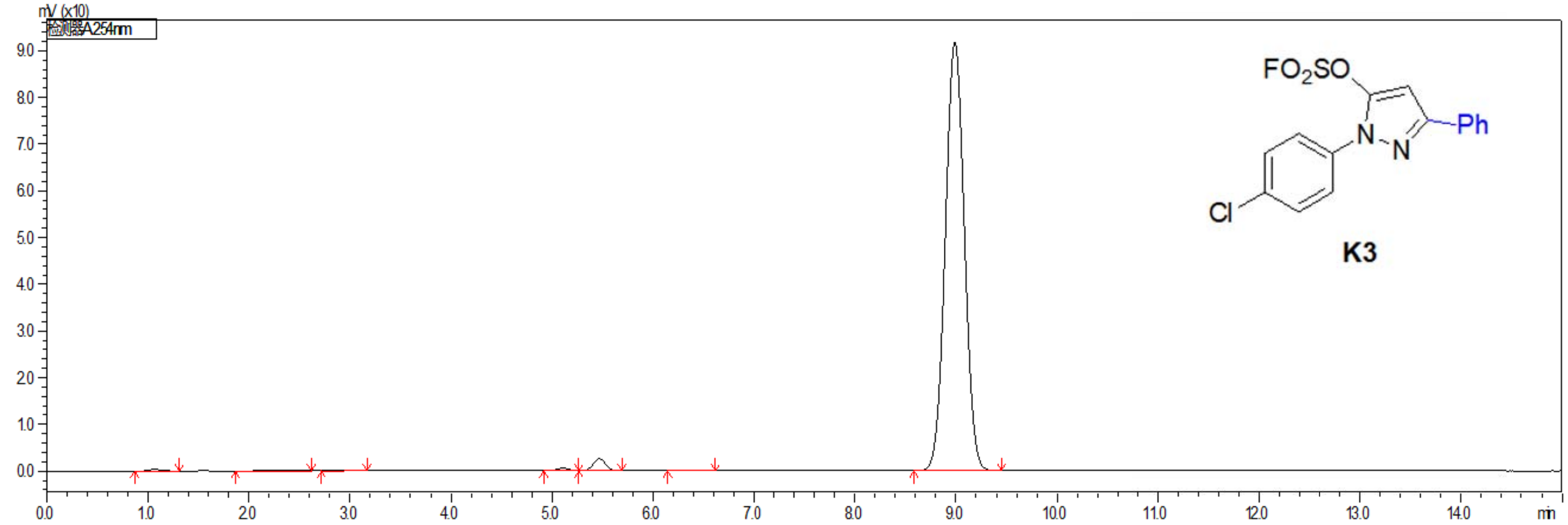

No	Ret Time(min)	Area(mAU*min)	Rel.Area(%)
1	1.061	3805	0.306
2	2.497	8029	0.645
3	2.803	3031	0.243
4	5.105	4471	0.359
5	5.468	21953	1.763
6	6.357	2136	0.172
7	8.989	1201506	96.512
Total		1244931	

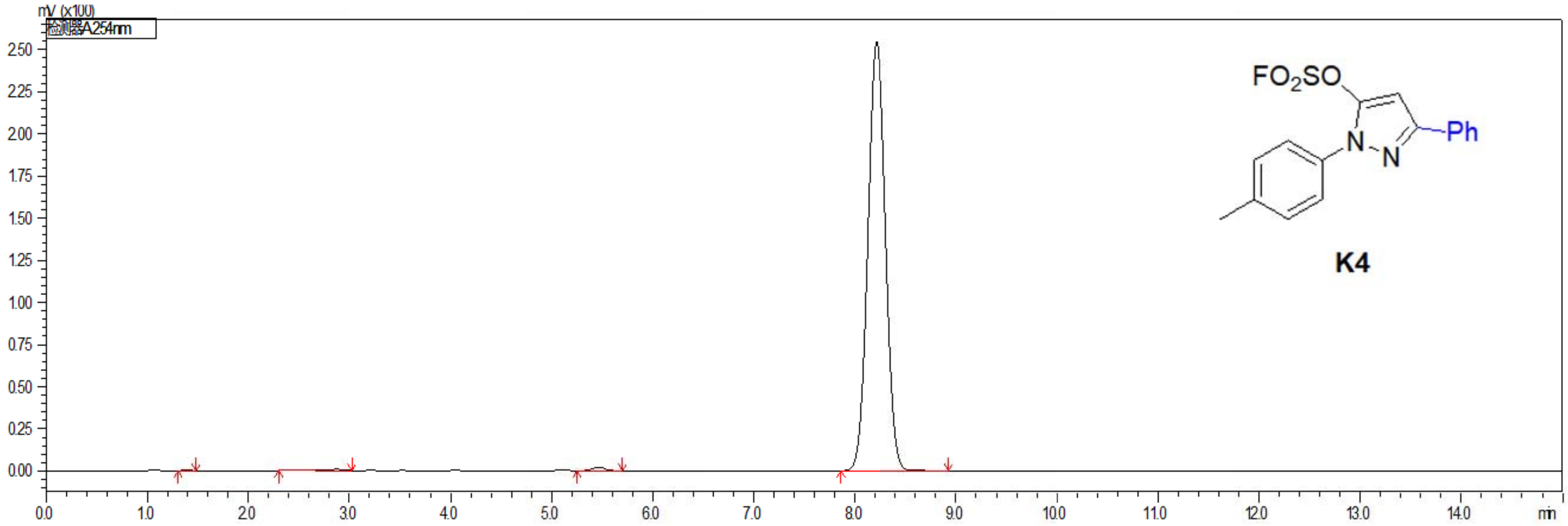

No	Ret Time(min)	Area(mAU*min)	Rel.Area(%)
1	1.378	4546	0.145
2	2.869	14158	0.452
3	5.463	19853	0.634
4	8.213	3093681	98.769
Total		3132239	

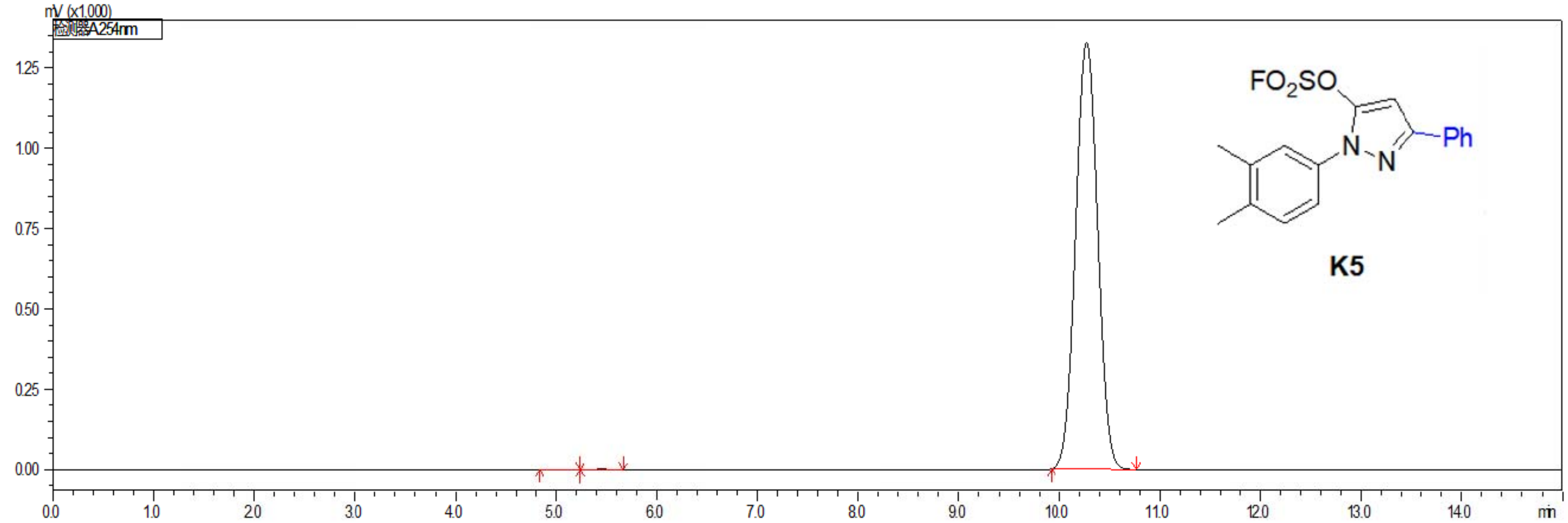

No	Ret Time(min)	Area(mAU*min)	Rel.Area(%)
1	5.09	3735	0.019
2	5.448	17759	0.088
3	10.274	20128695	99.893
Total		20150189	

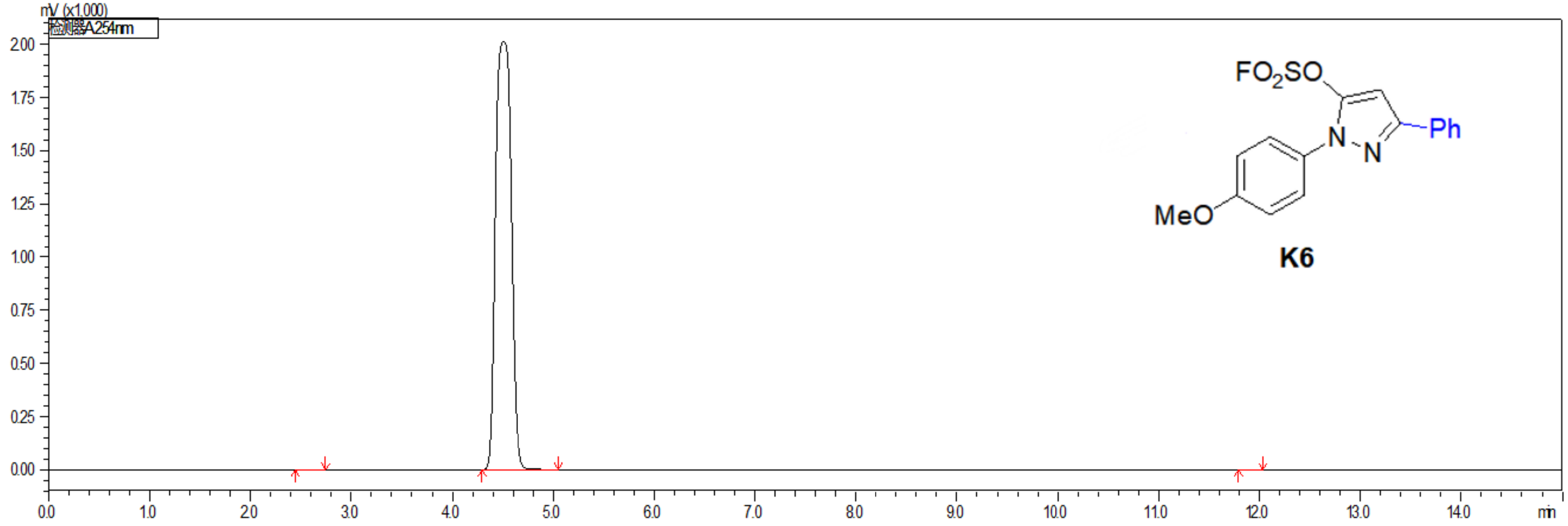

No	Ret Time(min)	Area(mAU*min)	Rel.Area(%)
1	2.63	4751	0.022
2	4.505	21723135	99.969
3	11.88	1918	0.009
Total		21729804	

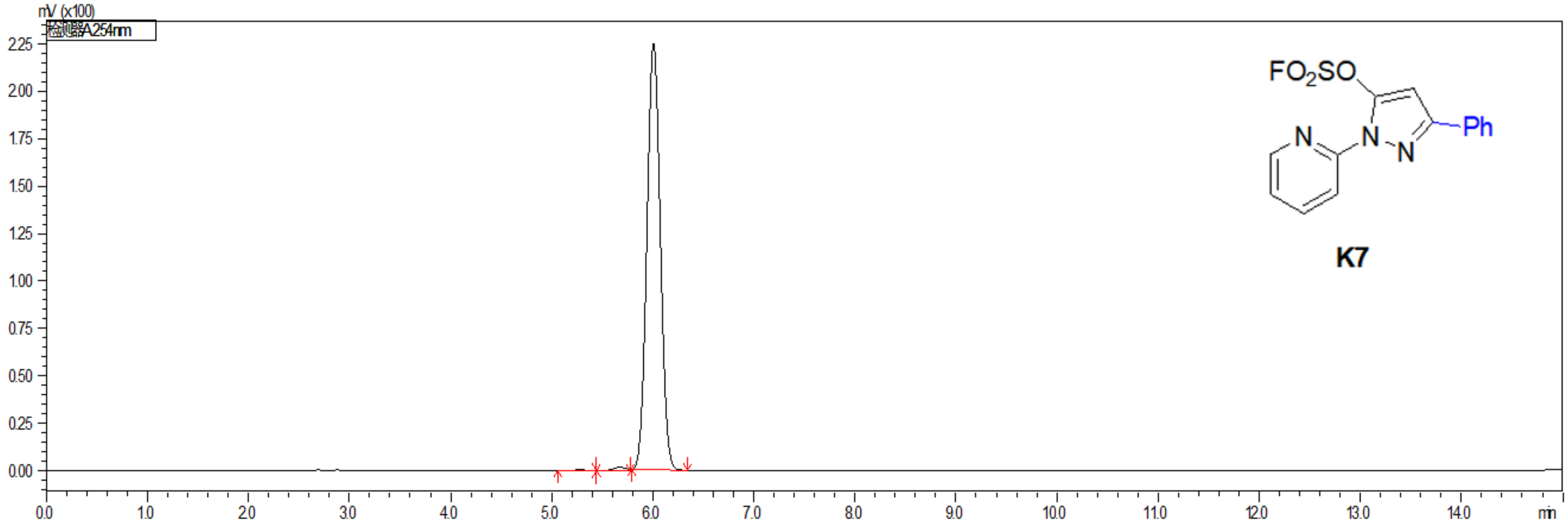

No	Ret Time(min)	Area(mAU*min)	Rel.Area(%)
1	5.282	3245	0.156
2	5.672	15663	0.751
3	6.006	2066086	99.093
Total		2084995	

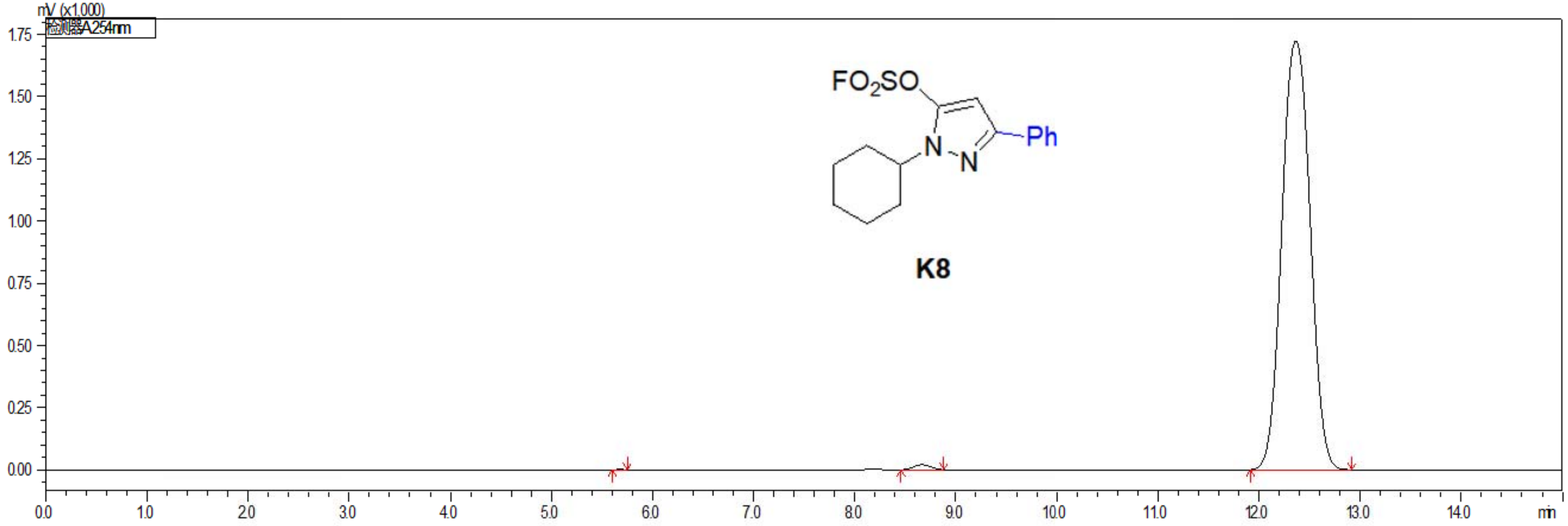

No	Ret Time(min)	Area(mAU*min)	Rel.Area(%)
1	5.68	6800	0.02
2	8.668	243714	0.725
3	12.364	33385467	99.255
Total		33635981	

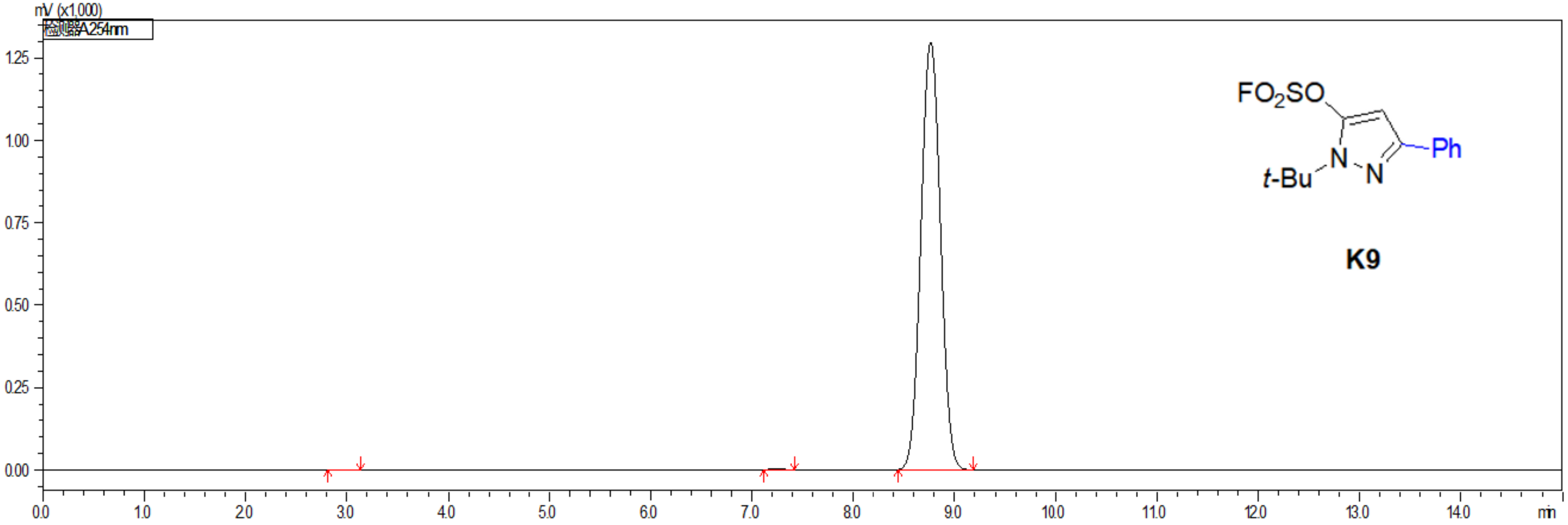

No	Ret Time(min)	Area(mAU*min)	Rel.Area(%)
1	2.869	6284	0.036
2	7.246	34357	0.197
3	8.763	17358850	99.766
Total		17399491	

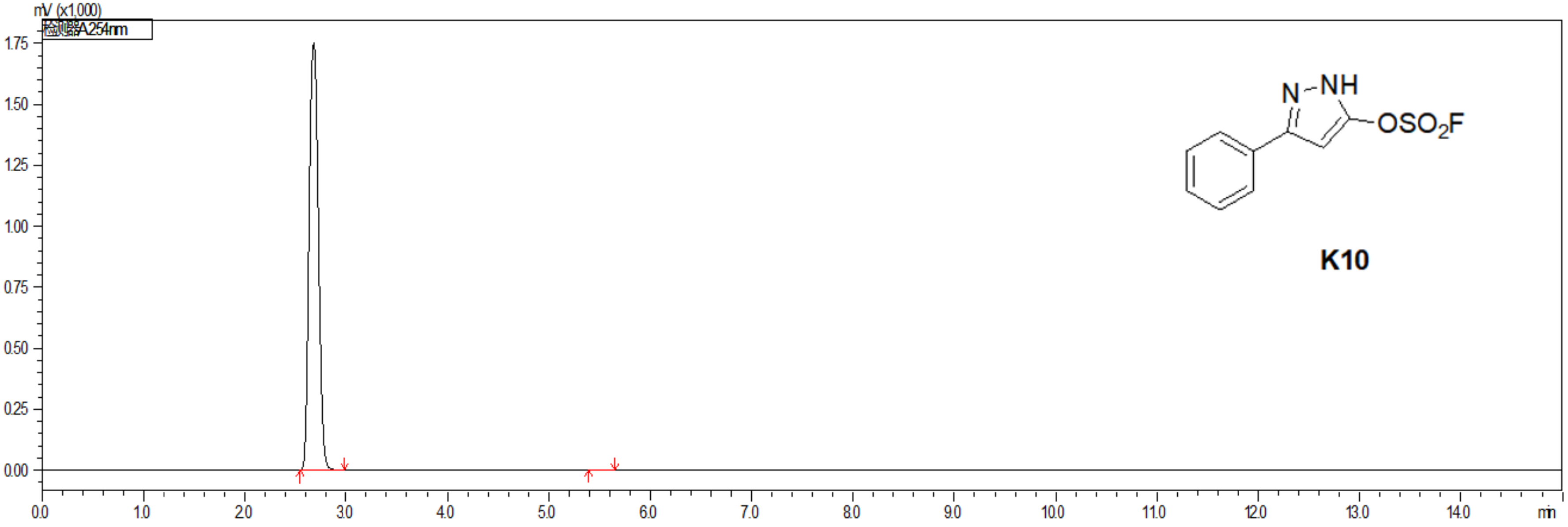

No	Ret Time(min)	Area(mAU*min)	Rel.Area(%)
1	2.675	11132024	99.9
2	5.486	11160	0.1
Total		11143184	

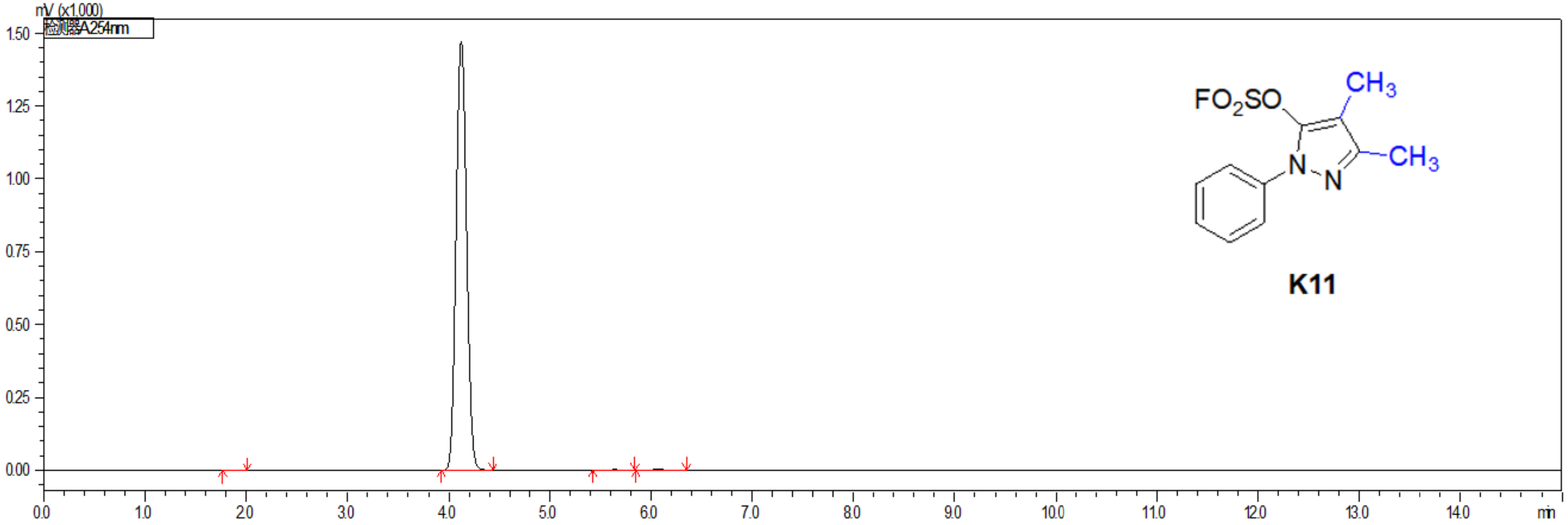

No	Ret Time(min)	Area(mAU*min)	Rel.Area(%)
1	1.85	5558	0.052
2	4.122	10579716	99.595
3	5.644	15832	0.149
4	6.072	21588	0.203
Total		10622695	

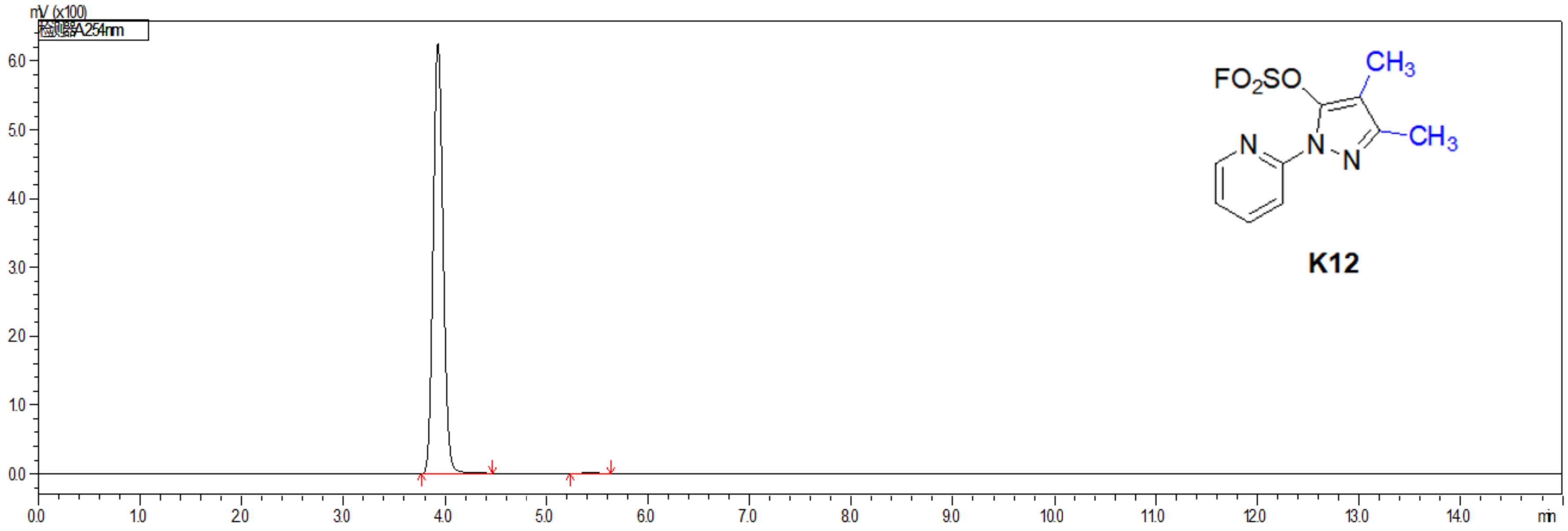

K12

No	Ret Time(min)	Area(mAU*min)	Rel.Area(%)
1	3.93	4234836	99.593
2	5.438	17288	0.407
Total		4252124	

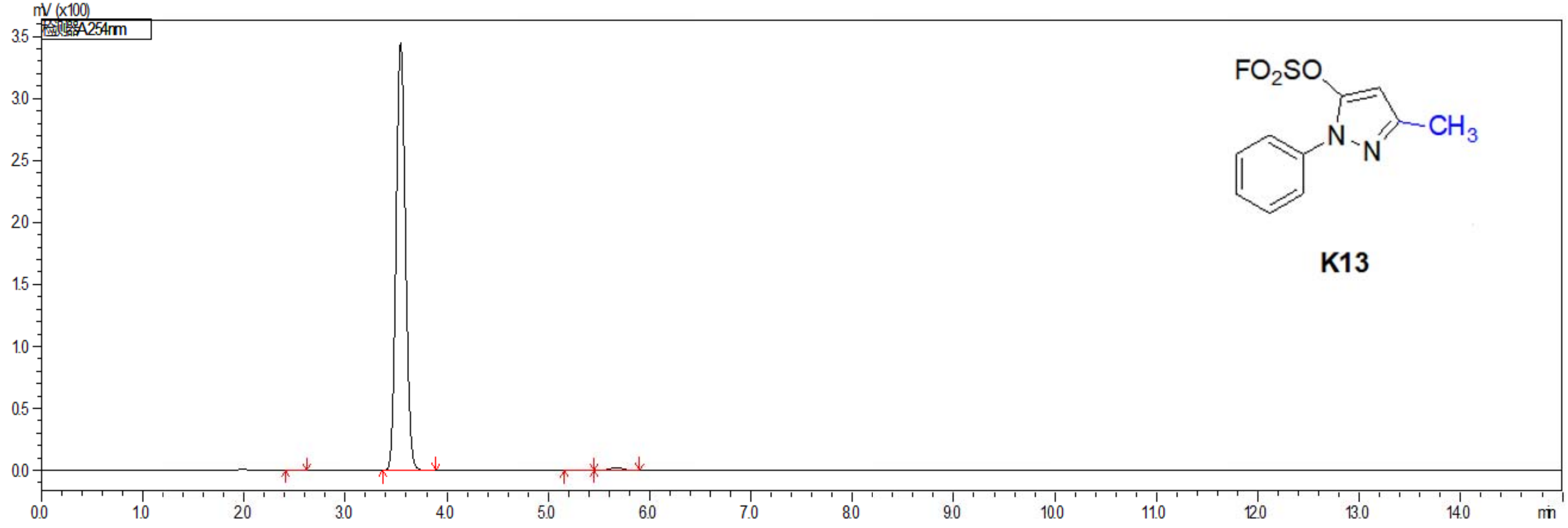

No	Ret Time(min)	Area(mAU*min)	Rel.Area(%)
1	2.5	1462	0.066
2	3.544	2202439	98.876
3	5.281	3469	0.156
4	5.671	20101	0.902
Total		2227471	

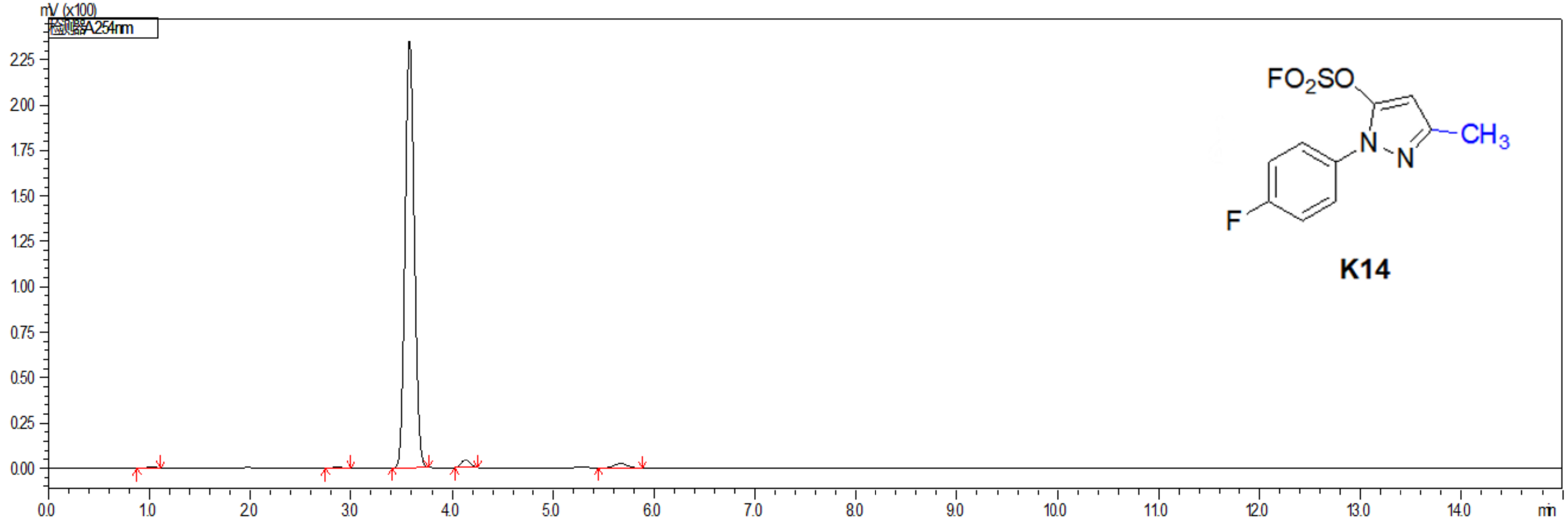

K14

No	Ret Time(min)	Area(mAU*min)	Rel.Area(%)
1	1.019	4216	0.272
2	2.871	2872	0.186
3	3.579	1489791	96.264
4	4.132	28294	1.828
5	5.67	22429	1.449
Total		1547602	

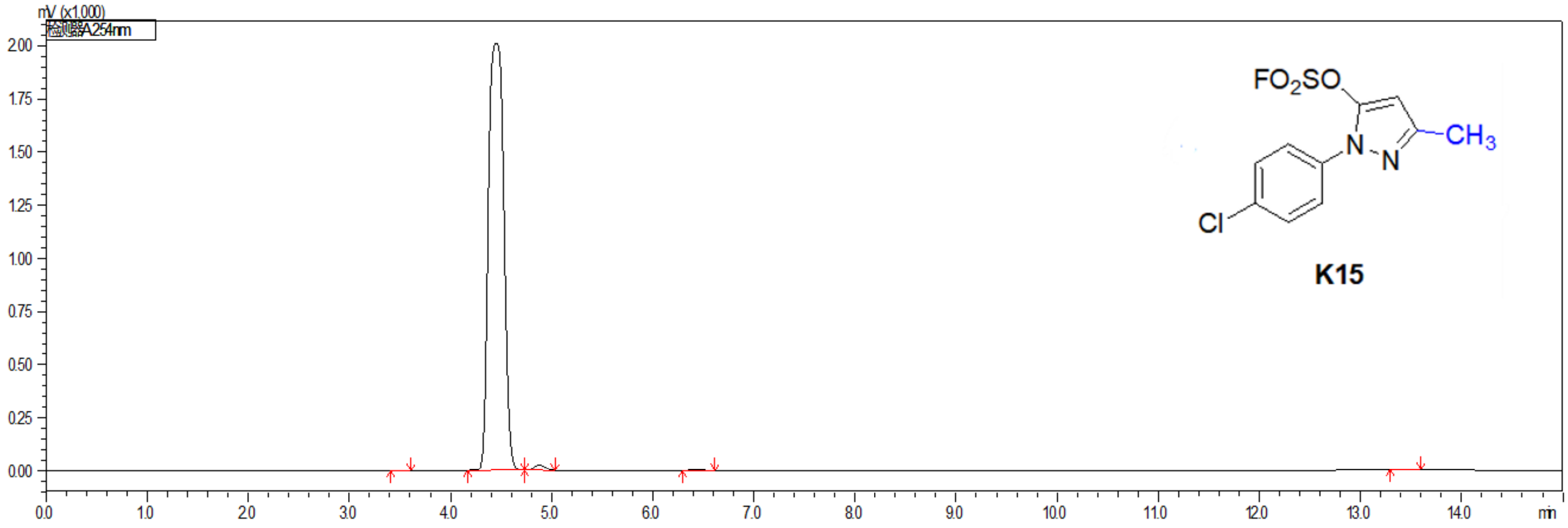

No	Ret Time(min)	Area(mAU*min)	Rel.Area(%)
1	3.514	10489	0.049
2	4.451	21061457	98.856
3	4.879	182484	0.857
4	6.435	46865	0.22
5	13.425	3883	0.018
Total		21305178	

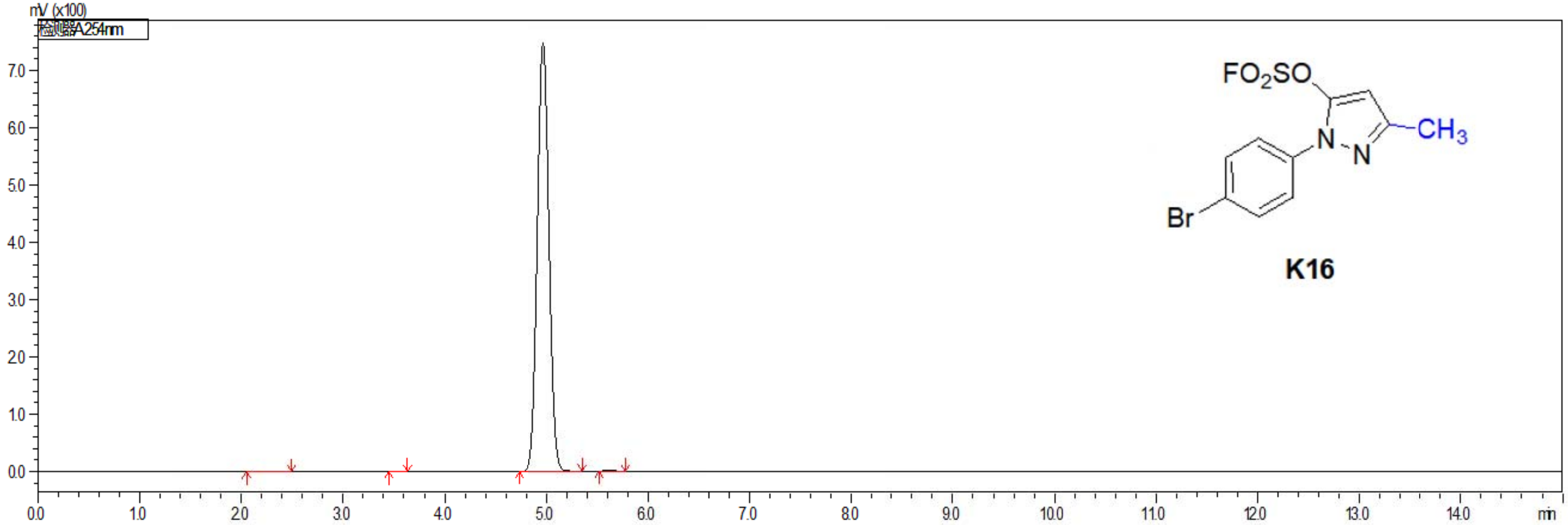

No	Ret Time(min)	Area(mAU*min)	Rel.Area(%)
1	2.266	5762	0.096
2	3.53	1826	0.031
3	4.967	5962424	99.674
4	5.615	11910	0.199
Total		5981922	

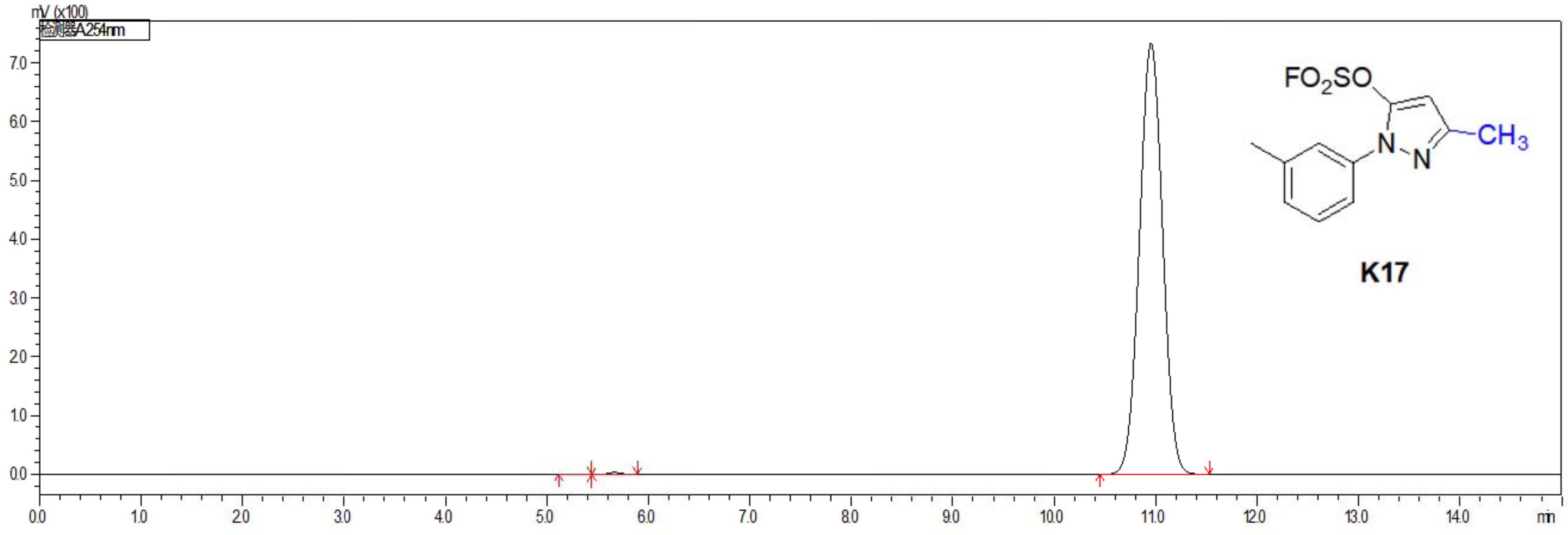

K17

No	Ret Time(min)	Area(mAU*min)	Rel.Area(%)
1	5.281	5079	0.044
2	5.67	26973	0.232
3	10.954	11598563	99.724
Total		11630615	

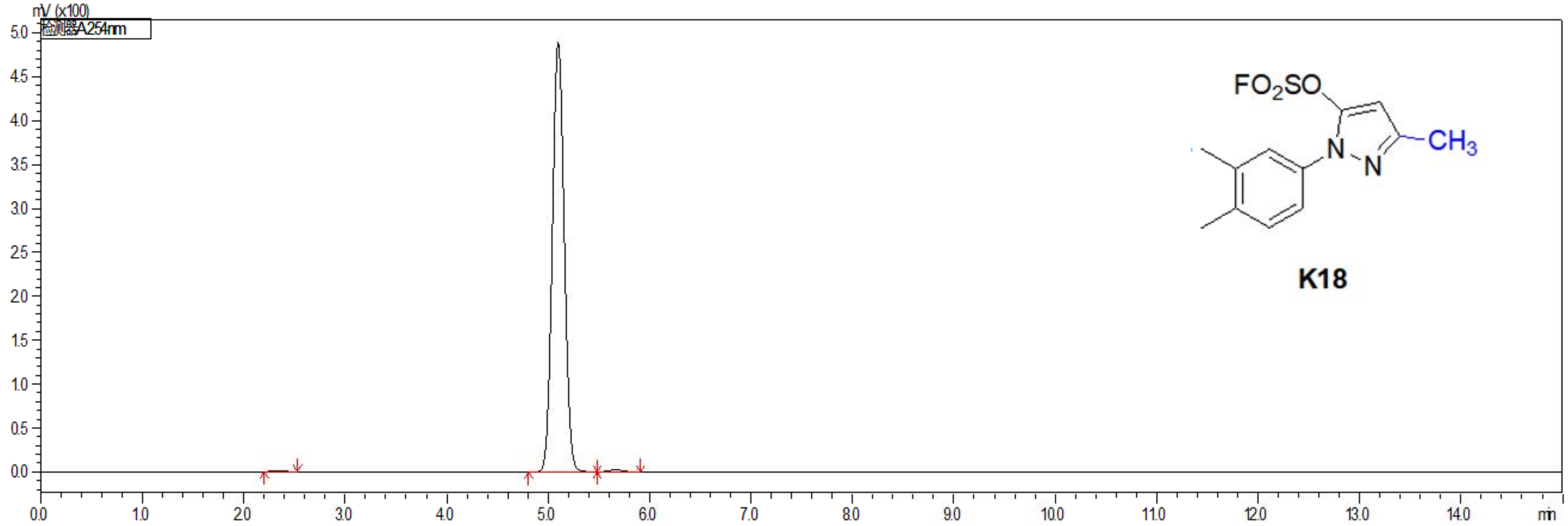

No	Ret Time(min)	Area(mAU*min)	Rel.Area(%)
1	2.333	4018	0.1
2	5.099	3968881	99.274
3	5.67	25023	0.626
Total		3997922	

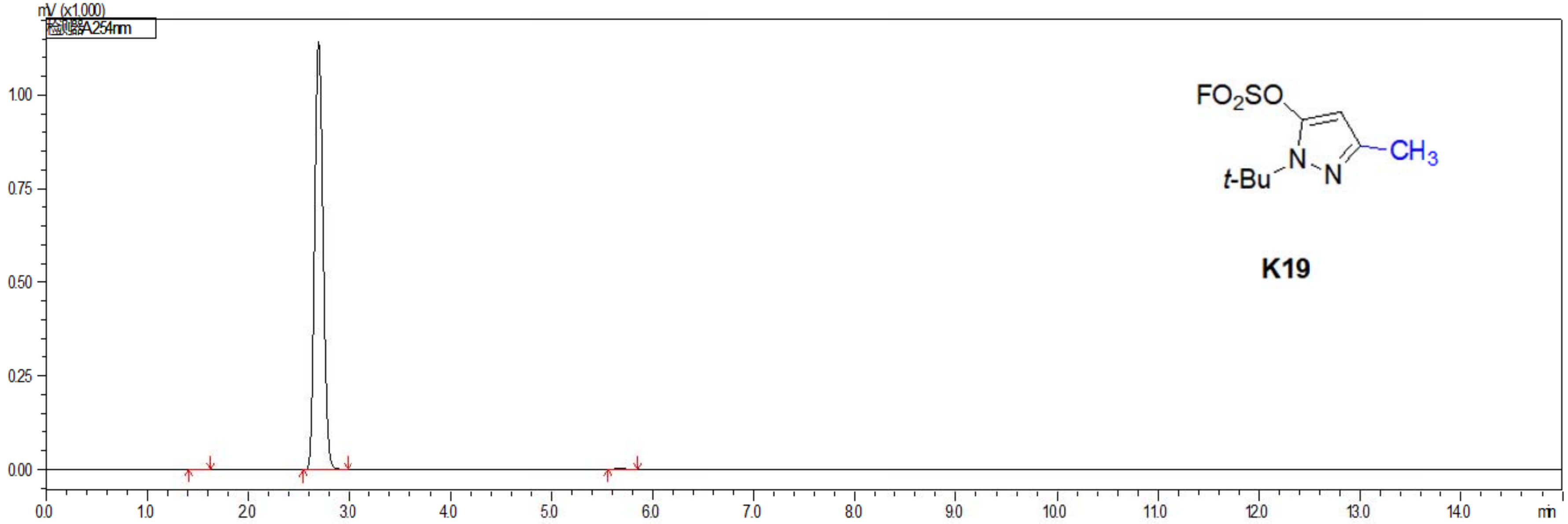

No	Ret Time(min)	Area(mAU*min)	Rel.Area(%)
1	1.54	1094	0.016
2	2.691	6633594	99.737
3	5.671	16385	0.246
Total		6651074	

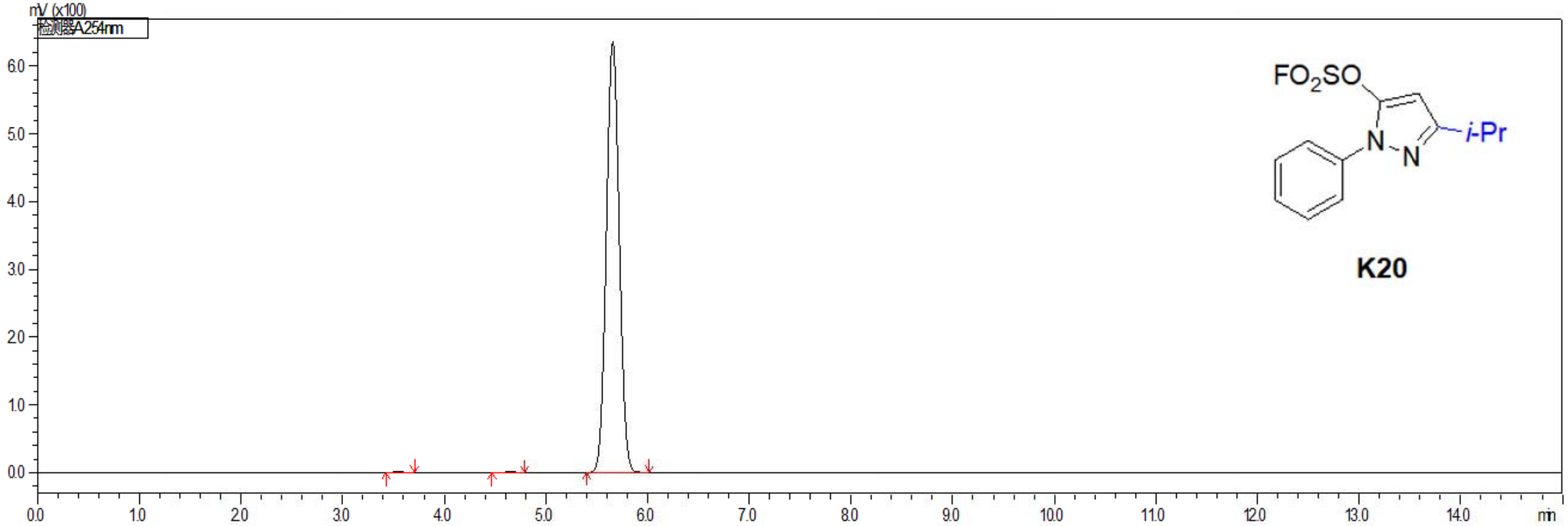

No	Ret Time(min)	Area(mAU*min)	Rel.Area(%)
1	3.542	7294	0.13
2	4.649	8976	0.16
3	5.657	5598533	99.71
Total		5614803	

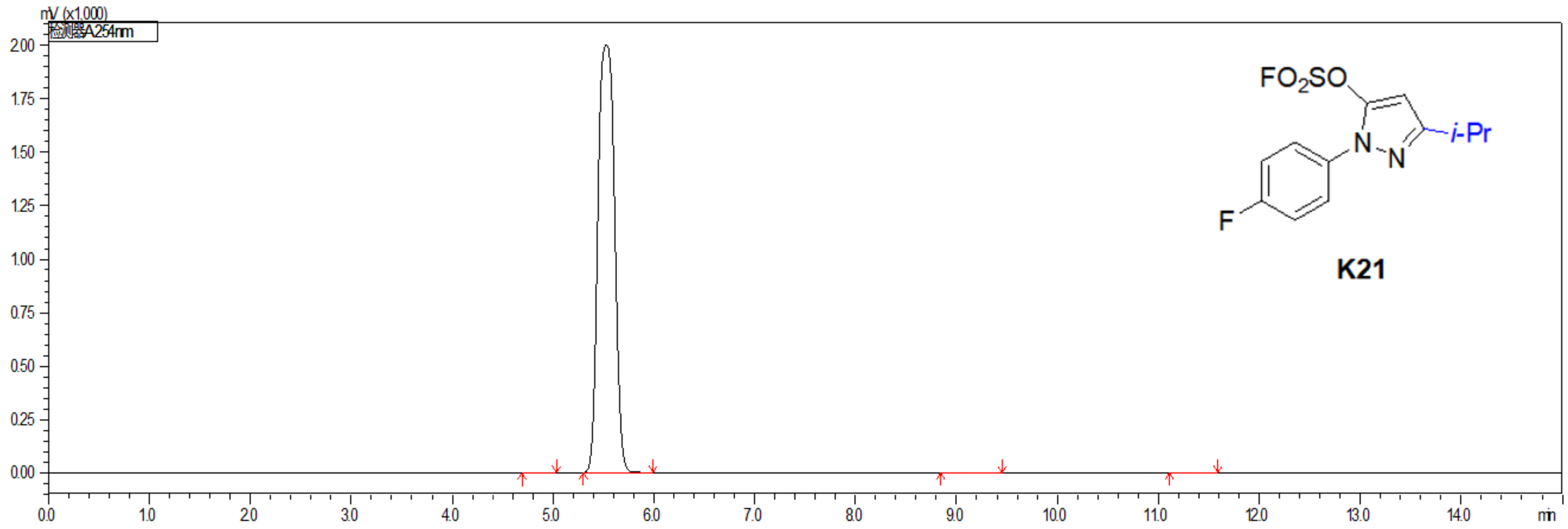

No	Ret Time(min)	Area(mAU*min)	Rel.Area(%)
1	4.847	11411	0.05
2	5.528	22841499	99.871
3	9.114	13227	0.058
4	11.303	4902	0.021
Total		22871039	

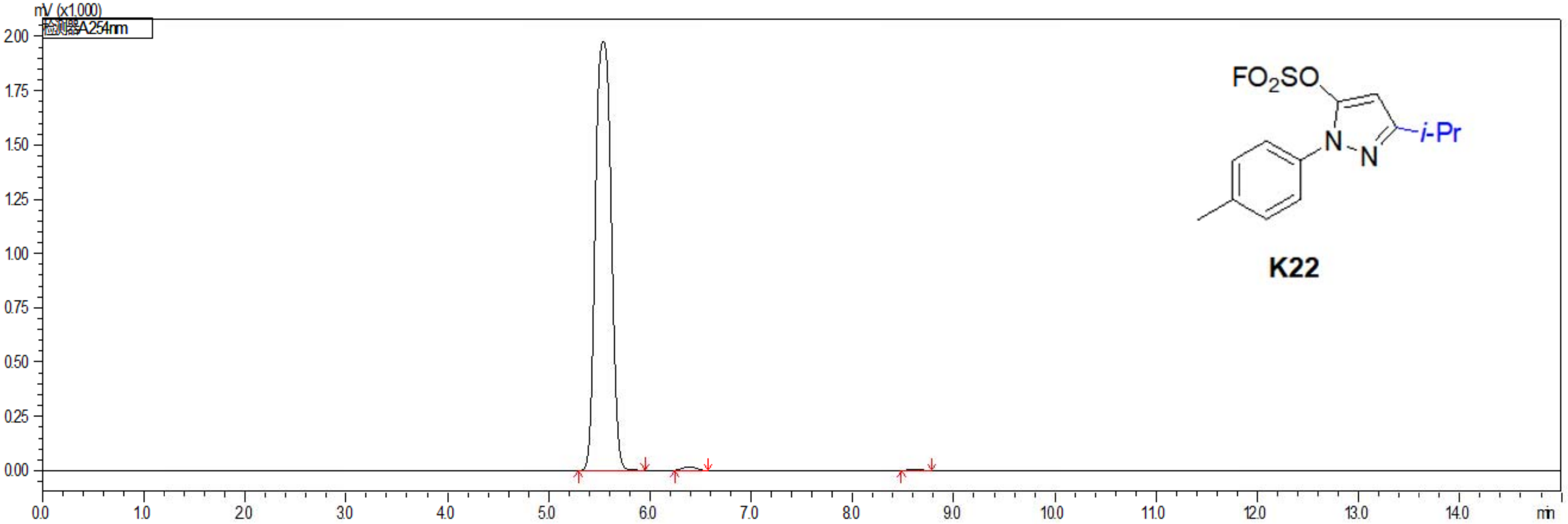

No	Ret Time(min)	Area(mAU*min)	Rel.Area(%)
1	5.539	21371061	99.171
2	6.388	148962	0.691
3	8.618	29767	0.138
Total		21549790	

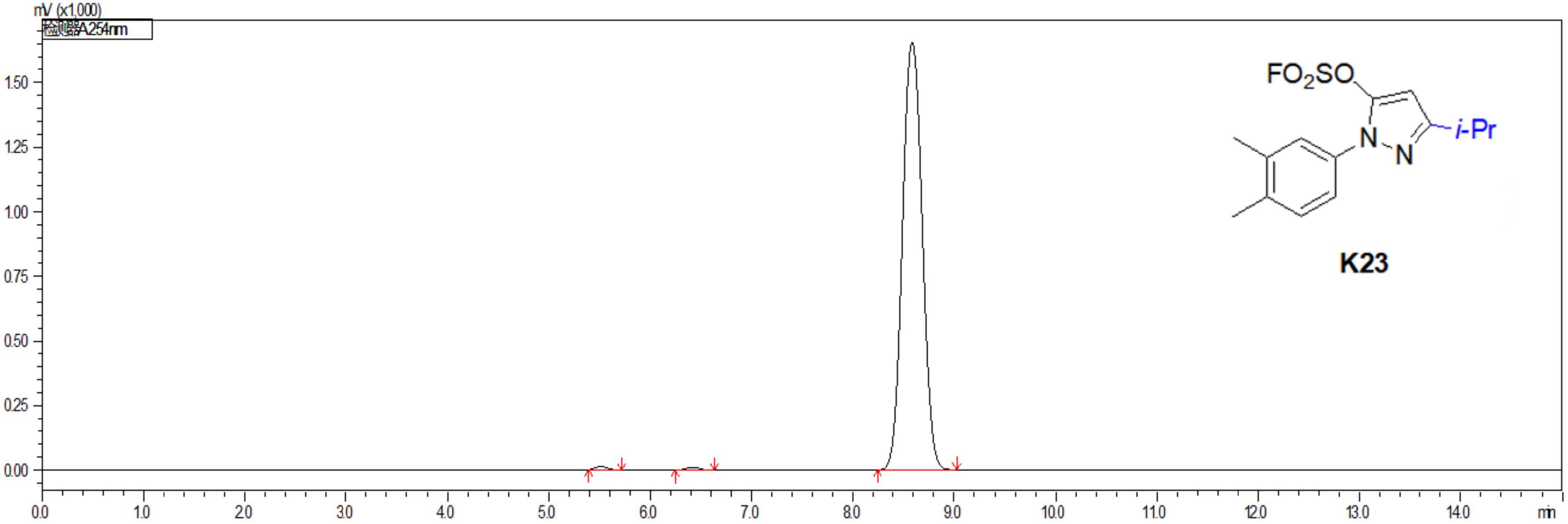

K23

No	Ret Time(min)	Area(mAU*min)	Rel.Area(%)
1	5.513	114076	0.506
2	6.415	106471	0.472
3	8.588	22325171	99.022
Total		22545718	

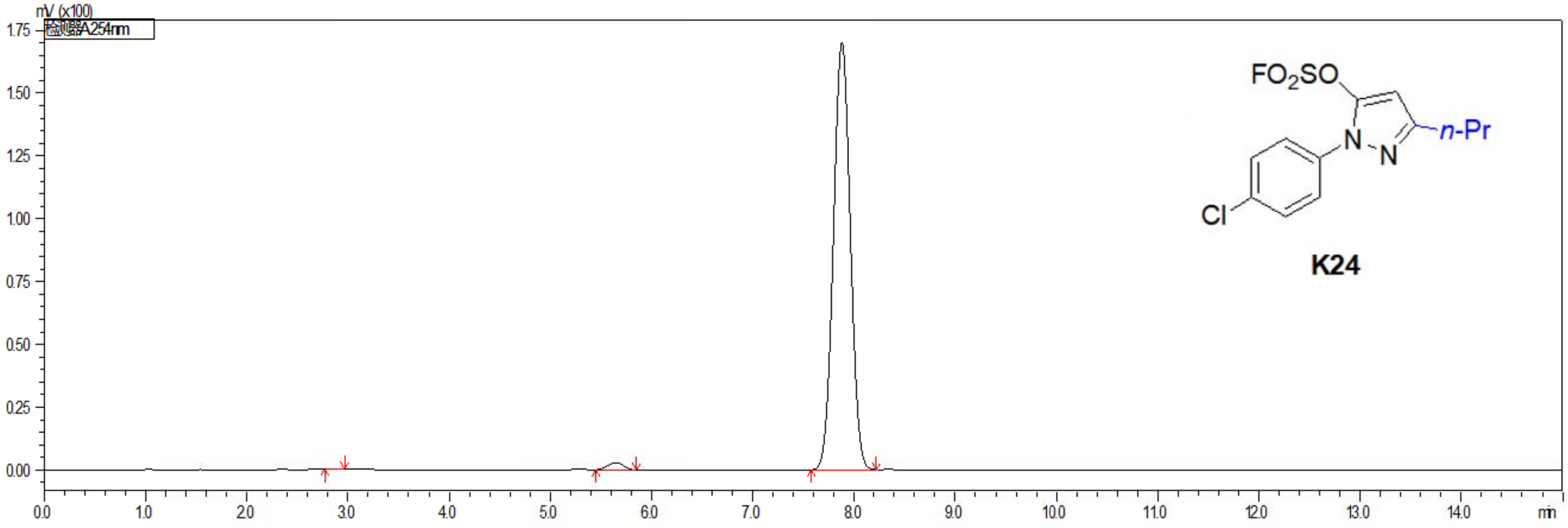

No	Ret Time(min)	Area(mAU*min)	Rel.Area(%)
1	2.872	1390	0.07
2	5.649	31324	1.567
3	7.877	1966680	98.364
Total		1999394	

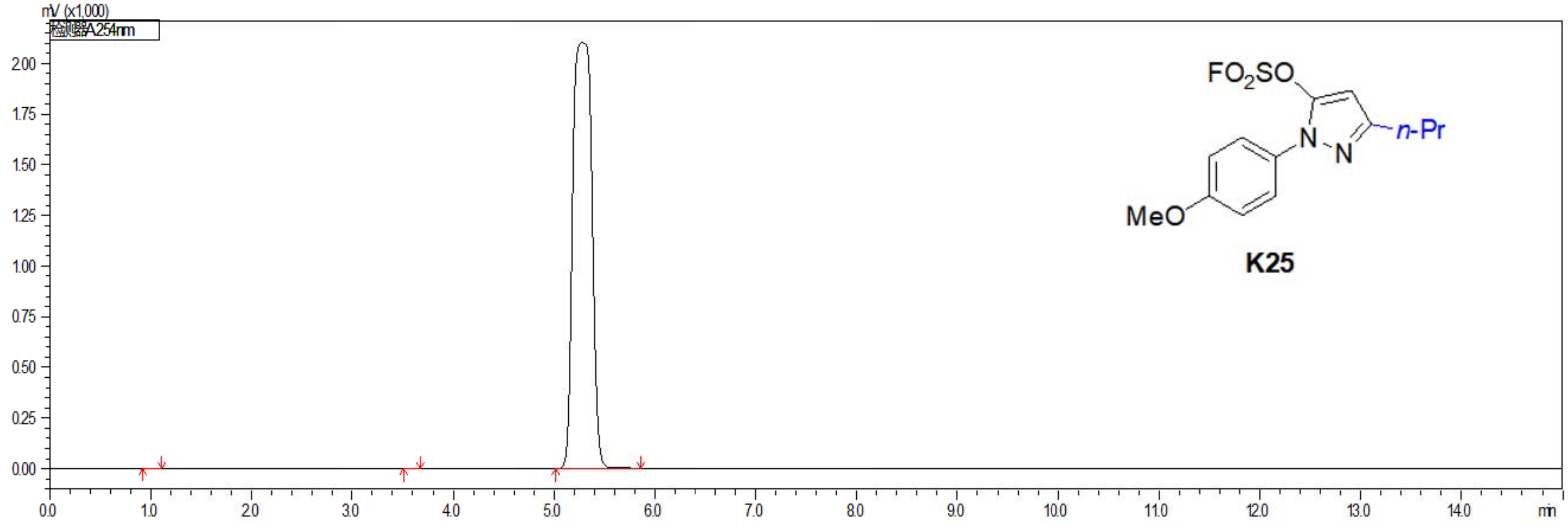

No	Ret Time(min)	Area(mAU*min)	Rel.Area(%)
1	1.02	5802	0.021
2	3.581	10243	0.037
3	5.279	27591290	99.942
Total		27607334	

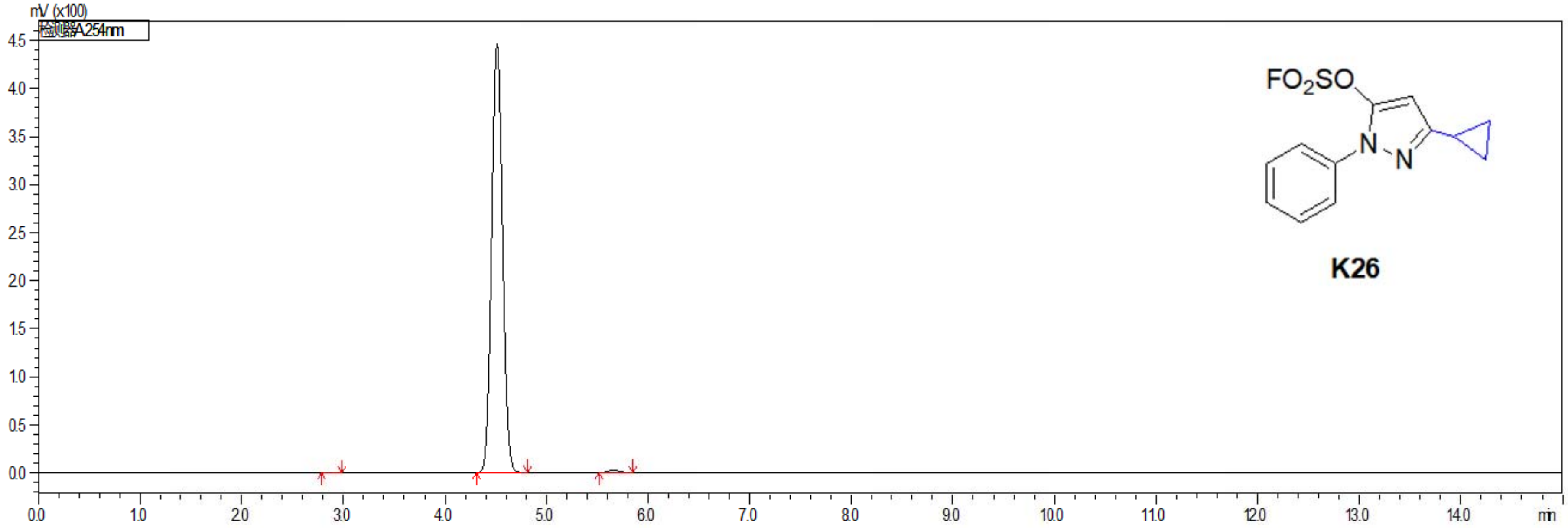

No	Ret Time(min)	Area(mAU*min)	Rel.Area(%)
1	2.864	1023	0.031
2	4.509	3327316	99.367
3	5.663	20174	0.602
Total		3348514	
